# Dual-filament regulation of relaxation in mammalian fast skeletal muscle

**DOI:** 10.1073/pnas.2416324122

**Published:** 2025-03-12

**Authors:** Cameron Hill, Michaeljohn Kalakoutis, Alice Arcidiacono, Flair Paradine Cullup, Yanhong Wang, Atsuki Fukutani, Theyencheri Narayanan, Elisabetta Brunello, Luca Fusi, Malcolm Irving

**Affiliations:** ^a^Randall Centre for Cell and Molecular Biophysics and British Heart Foundation Centre of Research Excellence, New Hunt’s House, Guy’s Campus, King’s College London, London SE1 1UL, United Kingdom; ^b^European Synchrotron Radiation Facility, Grenoble 38043, France; ^c^Centre for Human and Applied Physiological Sciences, Shepherd’s House, Guy’s Campus, King’s College London, London SE1 1UL, United Kingdom

**Keywords:** mammalian skeletal muscle, small-angle X-ray diffraction, actin, myosin, tropomyosin

## Abstract

Muscle contraction is driven by myosin motors from the thick filaments pulling on the actin-containing thin filaments of the sarcomere, and it is regulated by structural changes in both filaments. Thin filaments are activated by an increase in intracellular calcium concentration [Ca^2+^]_i_ and by myosin binding to actin. Thick filaments are activated by direct sensing of the filament load. However, these mechanisms cannot explain muscle relaxation when [Ca^2+^]_i_ decreases at high load and myosin motors are attached to actin. There is, therefore, a fundamental gap in our understanding of muscle relaxation, despite its importance for muscle function in vivo, for example, for rapid eye movements or, on slower timescales, for the efficient control of posture. Here, we used time-resolved small-angle X-ray diffraction (SAXD) to determine how muscle thin and thick filaments switch OFF in extensor digitorum longus (EDL) muscles of the mouse in response to decreases in either [Ca^2+^]_i_ or muscle load and to describe the distribution of muscle sarcomere lengths (SLs) during relaxation. We show that reducing load at high [Ca^2+^]_i_ is more effective in switching OFF both the thick and thin filaments than reducing [Ca^2+^]_i_ at high load in normal relaxation. In the latter case, the thick filaments initially remain fully ON, although the number of myosin motors bound to actin decreases and the force per attached motor increases. That initial slow phase of relaxation is abruptly terminated by yielding of one population of sarcomeres, triggering a redistribution of SLs that leads to the rapid completion of mechanical relaxation.

## Significance

The control of muscle contraction is usually described in terms of excitation–contraction coupling or muscle activation. Muscle relaxation has received much less attention, although its control is essential in the body for both rapid movements and efficient maintenance of posture. Relaxation is also impaired in conditions such as dystonia and Parkinson’s disease. Current models for muscle activation at the molecular level of the contractile filaments in muscle cannot explain relaxation. We filled this gap in current understanding at both the molecular and cellular levels, describing the roles of calcium- and load-dependent inactivation of muscle filaments and changes in local strain along the muscle fibers to determine the time course of relaxation.

The contraction of skeletal muscles is controlled by a combination of motor action potentials from the central nervous system and muscle-intrinsic or autoregulatory mechanisms that allow them to respond to local factors like muscle load and length. In vertebrates, motor action potentials are all-or-none “start” signals which coordinate the action of different motor units and muscle groups, and muscle relaxation is solely determined by muscle-intrinsic mechanisms. The speed and timing of muscle relaxation is a fundamental determinant of the efficient function of skeletal muscles in vivo. Rapid relaxation is essential for precise and rapid control of movements that require the coordinated action of different muscles, such as when playing a musical instrument, for example. Postural muscles, in contrast, benefit from slower relaxation to sustain tension at low metabolic cost. Muscle relaxation can also be impaired in diseases such as myotonic dystrophy and dystonia (1). However, despite the physiological and pathological significance of the control of muscle relaxation, the underlying mechanisms remain poorly understood.

At the molecular level, muscle contraction is driven by myosin motors from the thick filaments binding to actin sites in the thin filaments and pulling on them through a structural change in the actin-attached motor that is linked to ATP hydrolysis (2). The mechanical performance and metabolic cost of contraction are primarily determined by the availability of actin sites and myosin motors to participate in this interaction. The availability of actin sites is controlled by two other thin filament components: troponin and tropomyosin (3, 4). In resting muscle, when the intracellular calcium concentration [Ca^2+^]_i_ is low, these two proteins block the myosin-binding sites on actin (5–7). When a muscle cell is switched on by a motor action potential, Ca^2+^ is released from its internal stores and binds to troponin, triggering movements of troponin and tropomyosin that make actin sites available for myosin binding. Muscle relaxation is usually considered to be the reversal of this activating sequence following the reduction in [Ca^2+^]_i_ driven by active pumping of Ca^2+^ into their intracellular stores. However, the steric blocking model has an intrinsic directionality: When a myosin motor is bound to actin, the local tropomyosin cannot move back to its inhibitory position, a feature that suggests that myosin detachment, rather than the dissociation of calcium from troponin, may be the critical step controlling relaxation.

When the steric blocking model was first developed, it was assumed that myosin motors were always available for actin interaction. Subsequently, it became clear that most myosin motors are unavailable in resting muscle because they are folded back against the surface of the thick filament in an OFF conformation that inhibits actin interaction and ATP hydrolysis (8–10). Myosin motors leave this OFF state when the thin filament is activated by calcium, but the number of motors that do so depends on the load on the thick filament; when the load is very low, most motors stay in the OFF conformation, but when the load is high almost all the motors are available for actin interaction (11–15). This mechanosensing property of the thick filament contributes to the well-known force–velocity relationship and maximizes the metabolic efficiency of contraction. During fixed-end contraction, when the load becomes high, mechanosensing leads to a positive feedback loop, facilitating rapid activation (16).

The combination of the steric blocking model of thin filament activation and the mechanosensing hypothesis of thick filament activation leads to a fundamental paradox for muscle relaxation: How does muscle relax following electrical stimulation when the load is high, activating the thick filament, and when myosin motors are bound to actin, preventing the return of tropomyosin to its inhibitory conformation in the thin filament? The present experiments were carried out to address these questions and thereby clarify the mechanism of muscle relaxation. We used time-resolved small-angle X-ray diffraction (SAXD) to follow the activation states of both the thin and thick filaments during contractions of intact mouse extensor digitorum longus (EDL) muscles, which predominantly contain fast-twitch fibers, and in which we also controlled the load. We focused on two complementary questions: How do the activation states of the thick and thin filaments change when the load is removed at full calcium activation? How do they change when [Ca^2+^]_i_ decreases at the end of electrical stimulation at high load? The results show that reducing the load at high [Ca^2+^]_i_ is more effective in switching OFF both the thick and thin filaments than reducing [Ca^2+^]_i_ at high load, as in normal relaxation. In the latter case, the thick filaments initially remain fully ON, although the number of myosin motors bound to actin decreases and the force per attached motor increases. Filament inactivation and mechanical relaxation depend on a reproducible pattern of sarcomere length (SL) changes in which the single sarcomere population observed during isometric contraction is transiently replaced by two populations with different SLs, allowing muscles to escape from the positive feedback loop of high-load activation.

## Results

**Fig. 1.**
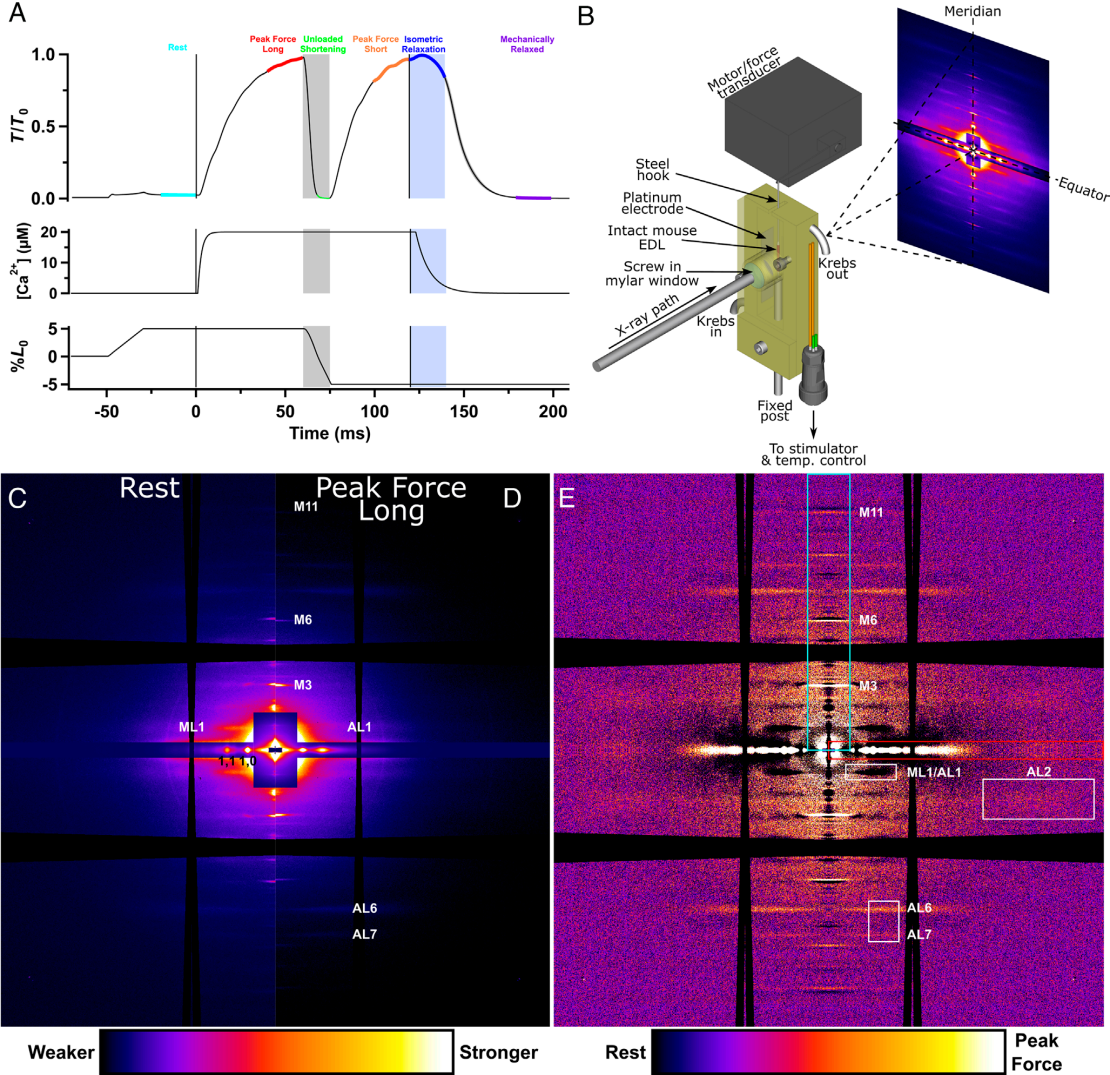
Mechanical protocol, experimental set-up and small-angle X-ray diffraction (SAXD) patterns. (*A*) Mechanical protocol. Force normalized to peak force (*T*/*T*_0_), the approximate time course of intracellular free calcium ion concentration [Ca^2+^]_i_ from (19), and applied change in muscle length as a percentage of optimal length (*L*_0_). Plateau force in fixed-end tetani at *L*_0_ was 270.6 ± 21.8 kPa (mean ± SEM; n = 7). Colored segments of the force trace denote periods used for averaging the SAXD data: cyan, rest; red, peak force at longer muscle length; green, unloaded shortening; orange, peak force at shorter muscle length; blue, isometric relaxation; purple, mechanically relaxed. The blue-shaded region denotes isometric relaxation. Vertical black lines mark the start (at time zero) and end of electrical stimulation. The dashed horizontal black line denotes zero force. (*B*) Arrangement for mounting muscles vertically on the synchrotron beamline with minimized X-ray path in solution, perfusion with oxygenated Krebs solution, electrical stimulation, force measurement, and control of muscle length. (*C*) *Left*-hand side of SAXD pattern from resting muscle with a sample-to-detector distance 2.0 m; average of ten frames from a total of 101 runs in three muscles, mirrored horizontally and vertically. Full beam equivalent exposure 381.8 ms. The broad black horizontal and vertical bands are produced by the gaps between the detector tiles and are fanned due to the alignment of the images from the slightly tilted muscles. (*D*) *Right*-hand side of pattern at peak force at the longer muscle length from the same runs as in (*C*). (*E*) Difference pattern obtained by subtracting the resting pattern (*C*) from the peak force pattern (*D*). Dark colors represent negative values in the difference image and, therefore, features that are stronger at rest. Cyan box: Integration region for meridional intensity distributions; the third (M3), sixth (M6), and eleventh (M11) myosin-based meridional reflections are labeled. Red box: Integration region for equatorial intensity distributions. White boxes: Integration regions for the second actin layer line (AL2) and the sixth and seventh actin layer lines (AL6 and AL7).

To separate the effects of intracellular calcium concentration [Ca^2+^]_i_ and filament stress on the activation states of the thick and thin filaments in skeletal muscle, we used a protocol that abolished force and filament stress while maintaining maximal calcium activation (17). We chose mouse EDL muscles stimulated repetitively at 28 °C for these experiments because the time course of [Ca^2+^]_i_ in these conditions has been characterized by previous studies. [Ca^2+^]_i_ increases rapidly after the first stimulus, peaking at about 2 ms as measured with a fast, low-affinity calcium indicator (18) (Fig. 1*A*). Peak [Ca^2+^]_i_ is about 20 µM, greatly exceeding the calcium dissociation constant of troponin, and troponin is expected to be more than half-saturated with calcium by 2 ms after the stimulus (19).

To measure the activation states of the thick and thin filaments in intact EDL muscles during contractions in which we also controlled the load, and recorded time-resolved SAXD patterns during the contractions (Fig. 1*B*).

Muscles were electrically stimulated at constant muscle length to develop high force for 60 ms Fig. 1*A*. Rapid shortening was imposed for 15 ms to reduce force to near zero, and then force was allowed to redevelop for 45 ms at the shorter length until the end of stimulation. [Ca^2+^]_i_ is maintained at a saturating level during stimulation and starts to fall soon after the last stimulus (18, 19) Fig. 1*A*. Force then decays rapidly at about 100 s^−1^ (*SI Appendix*, Table S1).

We determined the activation states of the thick and thin filaments during each phase of this protocol using SAXD (Fig. 1 *C*–*E*). The helical structures of the thick and thin filaments produce a series of “layer lines” in the diffraction pattern from resting muscle, which appear as horizontal lines for a vertically mounted muscle (Fig. 1*C*). The missing sections in the image are due to the gaps between the detector tiles. The thick filaments are responsible for the myosin-based “ML” layer lines that index on the roughly 43 nm fundamental axial repeat of the three-stranded helix of myosin monomers. During electrical stimulation at fixed length, the ML layer lines become much weaker and a new series of actin-based “AL” layer lines that index on the roughly 37 nm fundamental axial repeat of the two-stranded helix of actin monomers dominate the off-axial diffraction pattern (Fig. 1*D*). Both sets of layer lines can be visualized in a single image (Fig. 1*E*) by subtracting the resting SAXD pattern (Fig. 1*C*) from that recorded at peak force during the fixed-end phase of the contraction (Fig. 1*D*). The strongly diffracting features at rest, like the myosin-based layer lines, appear as darker colors in this difference map (Fig. 1*E*). The actin-based layer lines, in contrast, appear as lighter colors because they are stronger at peak force. The sharp myosin-based meridional reflections on the vertical axis of the image labeled M3, M6, and M11 in the cyan box are associated with the axial periodicity of the thick filament. Each of these reflections appears as a doublet composed of a lighter inner and darker outer line in the difference map, signaling the elongation of the thick filament on activation, which produces an X-ray reflection slightly closer to the center of the image. Finally, the bright reflections along the horizontal or equatorial axis of the diffraction pattern report interfilament separation and the mass distribution within the hexagonal lattice of thick and thin filaments.

### Thick Filament Regulation.

**Fig. 2.**
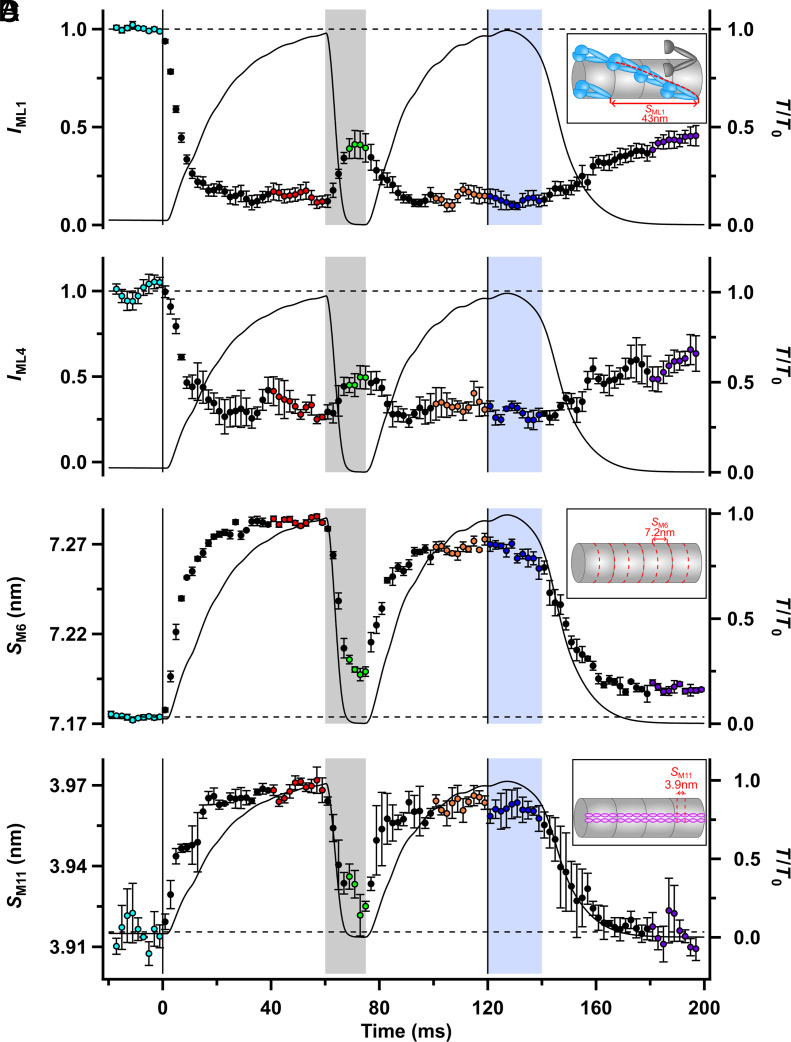
Structural signals associated with thick filament activation. (*A*) Intensity of the first myosin layer line (*I*_ML1_). *Inset*: Helical arrangement of pairs of myosin motors (blue) folded back against their tails that gives rise to the strong ML1 reflection with axial periodicity 43 nm in resting muscle; disordered motors are shown in gray. (*B*) Intensity of the fourth myosin layer line (*I*_ML4_). (*C*) Spacing of the sixth myosin-based meridional reflection (*S*_M6_) corresponding to an axial periodicity of 7.17 nm (*Inset*; red dashed lines) in resting muscle. (*D*) Spacing of the eleventh myosin-based meridional reflection (*S*_M11_) corresponding to the 3.9 nm axial periodicity of titin domains in the C-zone (*Inset*, magenta). Relative force (*T*/*T*_0_) is shown as the continuous black line. Color coding of the phases of the protocol is shown in Fig. 1*A*. Vertical lines denote the start and end of electrical stimulation; horizontal dashed lines denote the resting values of each parameter. Mean ± SEM for n = 4 muscles in (*A*) to (*C*), and n = 3 muscles in (*D*).

In resting muscle, myosin motors are folded back against their tails in a helical array with a periodicity of ca. 43 nm (Fig. 2*A*, *Inset*) that gives rise to a strong X-ray reflection called the first myosin-based layer line (ML1; Fig. 1*C*) (20) and its higher orders. The intensity of the ML1 reflection (*I*_ML1_; Fig. 2*A*) signals the activation state of the thick filament, and we used it to determine whether the thick filaments switch OFF when the load is reduced, or when [Ca^2+^]_i_ decreases during relaxation. ML1 partially overlaps the first actin-based layer line (AL1) associated with the ca. 37 nm helical periodicity of the thin filament (*SI Appendix*, Fig. S1*B*), but ML1 and AL1 can be separated by Gaussian deconvolution under the assumption that their axial periodicities *S*_ML1_ and *S*_AL1_ are constant. The best fit to the present data gave *S*_ML1_ = 42.6 ± 0.1 nm and *S*_AL1_ = 36.7 ± 0.1 nm (mean ± SEM).

The intensity of the ML1 reflection (*I*_ML1_) decreased rapidly following electrical stimulation in fixed-end conditions (Fig. 2*A*, red) to about 15% of its resting value. The half-time for the change in the amplitude of the ML1 reflection (*A*_ML1_; the square root of *I*_ML1_, the preferred metric for the associated mass transfer) was only 5.6 ± 0.5 ms, much less than that of force development, 16.9 ± 0.4 ms (*SI Appendix*, Table S1). *I*_ML1_ recovered rapidly but partially during unloaded shortening (Fig. 2*A*, green), indicating reformation of the resting helical array of myosin motors with a half-time of only 4.7 ± 0.4 ms for *A*_ML1_, close to that of the accompanying force decrease, 3.9 ± 0.2 ms. At the end of shortening, *A*_ML1_ recovered with a half-time of 6.7 ± 1.6 ms. There was no detectable change in *I*_ML1_ during isometric relaxation (blue) and only partial recovery during mechanical relaxation (21); the value of *I*_ML1_ in the mechanically relaxed muscle (purple) was similar to that at the end of unloaded shortening (green; *SI Appendix*, Table S2). These results reveal a striking difference between the rapid partial recovery of the folded OFF state of the myosin motors triggered by removing the load at full calcium activation, which follows the force decrease in the first few milliseconds, and the very slow recovery that follows the decline of [Ca^2+^]_i_ following the end of electrical stimulation.

To check whether these results might have been influenced by the close proximity of the AL1 and ML1 layer lines, we determined the time course of the intensity of the fourth myosin layer line (*I*_ML4_; Fig. 2*B* and *SI Appendix*, Fig. S1*C*), which is well separated from any actin-based layer line. ML4 is much weaker than ML1, so *I*_ML4_ is noisier than *I*_ML1_, but the two time courses were similar within the available resolution, confirming the above conclusions.

The spacing of the sixth myosin-based meridional reflection (*S*_M6_) has been used extensively as a measure of the axial periodicity of the thick filament and increases by about 1.5% when the thick filament is activated (Fig. 2*C* and *SI Appendix*, Table S2 and Fig. S1G) (21–23). *S*_M6_ therefore provides an additional X-ray signal for the activation state of the thick filament. The structural basis of the M6 X-ray reflection is unknown, but its periodicity (*S*_M6_) is about 7.2 nm, corresponding to half the axial distance between the layers of myosin motors (Fig. 2*C*, *Inset*). *S*_M6_ increased with a half-time of 5.2 ± 0.2 ms at the start of stimulation (*SI Appendix*, Table S1), almost the same as that of *A*_ML1_; both these structural signals of thick filament activation are significantly faster than force development. *S*_M6_ decreased during unloaded shortening with a half-time of 4.8 ± 0.2 ms and recovered rapidly during force redevelopment at the shorter length (orange), again following *A*_ML1_ in both periods. *S*_M6_ decreased only slightly during isometric relaxation and did not fully recover its value after mechanical relaxation (21), so that like *A*_ML1_, its value during mechanical relaxation (purple) was similar to that at the end of unloaded shortening (green). In summary, the time course of the change in the axial periodicity of the thick filament signaled by *S*_M6_ in this protocol follows its helical order signaled by *A*_ML1_.

The ca. 3.9 nm spacing of the eleventh myosin-based meridional X-ray reflection (*S*_M11_; Fig. 2*D*) corresponds to the domain repeat of the scaffold protein titin in the region of the thick filament containing myosin binding protein-C, the C-zone (9, 24) (Fig. 2*D*, *Inset*). *S*_M11_ increased by about 1.4% during force development with the same half-time as *S*_M6_ (*SI Appendix*, Tables S1 and S2), within the limits of the signal-to-noise. *S*_M11_ and *S*_M6_ had an equally rapid response to the imposition of unloaded shortening (green; *SI Appendix*, Table S1), but in contrast to *S*_M6_, *S*_M11_ recovered fully to its resting level during mechanical relaxation (purple).

### Thin Filament Regulation.

**Fig. 3.**
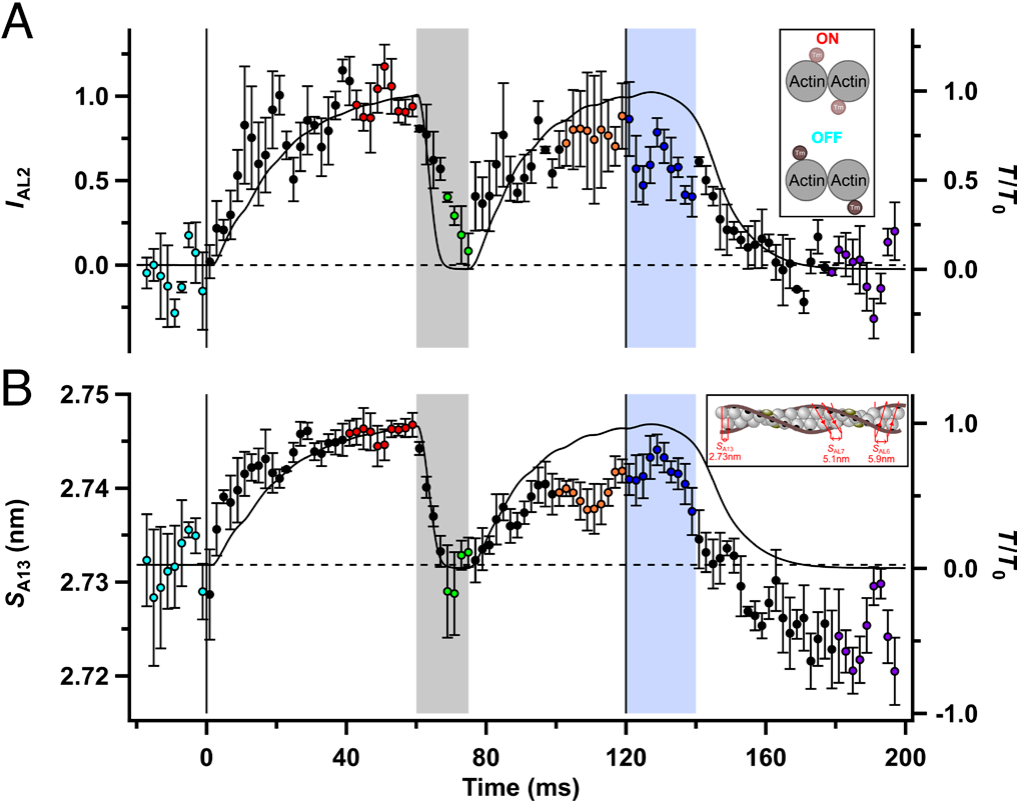
Structural signals associated with thin filament activation. (*A*) Intensity of the second actin layer line (*I*_AL2_). *Inset*: Schematic transverse section through the thin filament indicating azimuthal positions of tropomyosin (Tm) at rest (cyan; dark brown circles) and in active contraction (red; light brown circles). (*B*) Spacing of the thirteenth meridional actin-based reflection (*S*_A13_), calculated from the spacings of the sixth and seventh actin layer lines. *Inset*: Longitudinal thin filament schematic with ca 2.73 nm axial repeat of actin monomers associated with the A13 reflection, and genetic actin helices associated with the AL6 (5.9 nm) and AL7 (5.1 nm) reflections. Tropomyosin (dark brown) blocks myosin binding sites on actin (black) when troponin (dark purple) is not saturated by Ca^2+^. Relative force (*T*/*T*_0_) is shown as the continuous black line. Color coding of the phases of the protocol is shown in Fig. 1*A*. Vertical lines denote the start and end of electrical stimulation; horizontal dashed lines denote the resting values of each parameter. Mean ± SEM for n = 3 muscles in (*A*) and n = 4 muscles in (*B*).

The actin-based AL2 layer line (Fig. 1*E*) is associated with the activated state of the thin filament, in which tropomyosin has moved azimuthally toward the center of the groove between the two strands of the actin helix, uncovering the myosin binding sites on actin (5, 25, 26) (Fig. 3*A*, *Inset*). The intensity of the AL2 reflection (*I*_AL2_) therefore provides an X-ray signal for the activation state of the thin filament, and we used it to determine how thin filament activation changes in response to a decrease in load at full calcium activation, or the decrease in [Ca^2+^]_i_ during relaxation. *I*_AL2_ was very low in resting muscle (Fig. 3*A*; cyan) but increased during fixed-end contraction (red), with a time course similar to that of force development. *I*_AL2_ decreased during unloaded shortening (green), almost regaining its resting level by the end of the shortening ramp, and recovered during force redevelopment after unloaded shortening. *I*_AL2_ decreased by only about 20% during isometric relaxation (*SI Appendix*, Table S2) but fully recovered its value after mechanical relaxation (purple). These results show that thin filament activation is strongly dependent on active force as well as on intracellular calcium concentration.

Analogous to the elongation of the thick filament on activation described in the preceding section, the axial periodicity of actin monomers, usually called *S*_A13_ in reference to a model of the thin filament with exactly 13 turns in each ca. 37 nm helical repeat (Fig. 3*B*, *Inset*), also increases by about 0.4% during fixed-end force development (27–29). Part of this increase is due to the compliance of the thin filament (27, 30, 31), but a larger component seems to be associated with thin filament activation per se (27, 29). Here, we determined *S*_A13_ from the changes in the spacings of the AL6 and AL7 layer lines (Fig. 1*E* and *SI Appendix*, Table S2 and Fig. S1 D and *E*), which are described in more detail in the following section. We found that *S*_A13_ increased by 0.5% during activation at fixed length but recovered its resting value during unloaded shortening (Fig. 3*B* and *SI Appendix*, Table S2), consistent with the presence of a force-dependent component of thin filament activation as inferred above from *I*_AL2_, although quantitative interpretation is complicated by the low signal-to-noise ratio and the maintained undershoot of *S*_A13_ following mechanical relaxation. The increase of *S*_A13_ at the start of stimulation also seems to be faster than force (Fig. 3*B* and *SI Appendix*, Table S1).

### Movement of Myosin Motors.

**Fig. 4.**
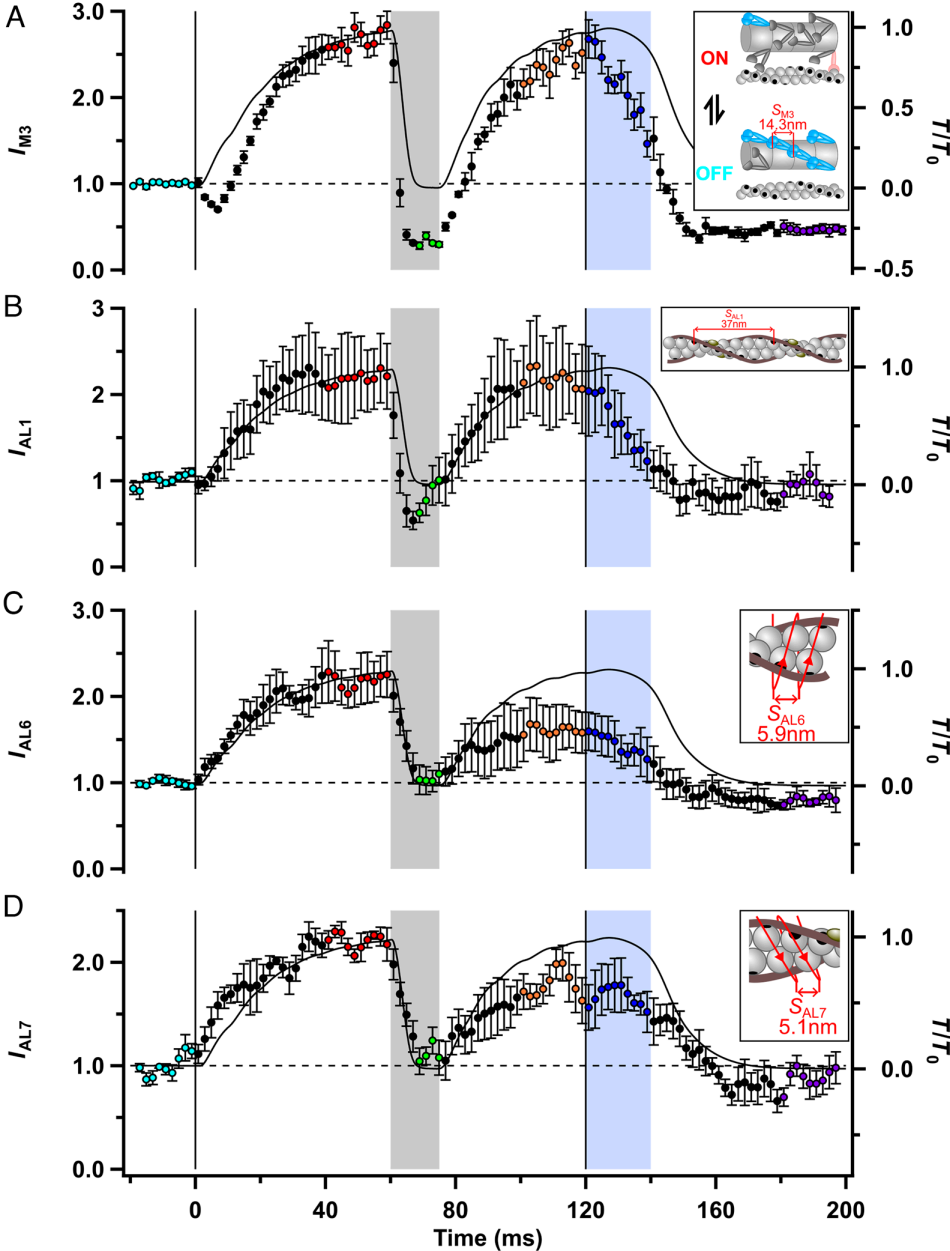
Structural signals of myosin motor conformation and actin attachment. (*A*) Intensity of the third myosin-based meridional reflection (*I*_M3_). *Inset*: Schematic of overlapping thin and thick filaments with myosin motors in their resting (blue; axial periodicity 14.34 nm), actin-attached force-generating (light red), and disordered (dark gray) conformations. (*B*) Intensity of the first actin layer line (*I*_AL1_). *Inset*: Thin filament schematic showing the ca 37 nm helical periodicity associated with the AL1 reflection. (*C*) Intensity of the sixth actin layer line (*I*_AL6_) corresponding to the genetic helix with a periodicity of 5.9 nm (*Inset*). (*D*) Intensity of the seventh actin layer (*I*_AL7_) corresponding to the genetic helix with a periodicity of 5.1 nm (*Inset*). Relative force (*T*/*T*_0_) is shown as the continuous black line. Color coding of the phases of the protocol is shown in Fig. 1*A*. Vertical lines denote the start and end of electrical stimulation; horizontal dashed lines denote the resting values of each parameter. Mean ± SEM for n = 4 muscles (*A*), (*C*), and (*D*) and n = 7 muscles for (*B*).

At the start of electrical stimulation in fixed-end conditions, myosin motors leave the helically ordered OFF state, bind to actin, and generate force. The structural changes in the motors associated with those processes can be monitored using the M3 meridional reflection (Fig. 1 *C*–*E* and *SI Appendix*, Fig. S1*F*) associated with the roughly 14.3 nm axial periodicity of the motors along the thick filament (Fig. 4*A*, *Inset*). The intensity of the M3 reflection (*I*_M3_, Fig. 4*A*) decreases transiently at the start of stimulation as the OFF state is lost, then increases to about 2.5 times its resting level (red) as force-generating actin-attached motors are formed (21, 32) (*SI Appendix*, Table S2). *I*_M3_ is sensitive to the activation state of the thick filament, like *I*_ML1_, but also signals the number and conformation of force-generating motors. All these parameters are likely to change during activation. *I*_M3_ decreased rapidly at the start of unloaded shortening, signaling tilting of myosin motors during the working stroke (33) followed by detachment, and for most of the shortening period (green), *I*_M3_ was much lower than at rest (34). This maintained low level is expected from the very low fraction of motors attached to actin during unloaded shortening (35) and the incomplete reformation of the helical OFF state (Fig. 2*A*). *I*_M3_ recovered during force redevelopment (Fig. 4*A*, orange) and decreased dramatically during isometric relaxation (blue) despite the maintained high level of force. These results show that the number of actin-attached motors decreases substantially during isometric relaxation, implying an increase in the force per attached motor (36), despite the fact that the thick filaments remain ON as signaled by *I*_ML1_. *I*_M3_ in mechanically relaxed muscle (purple) was similar to that during unloaded shortening (green) and lower than that at rest (cyan), consistent with the incomplete recovery of the OFF state of the thick filament in mechanically relaxed muscle described above.

The intensities of the actin-based layer lines signal the number and conformation of myosin motors attached to actin (37). The first actin layer line (AL1) is associated with the ca. 37 nm helical repeat of the thin filaments (Fig. 4*B*, *Inset*). Its intensity (*I*_AL1_; Fig. 4*B* and *SI Appendix*, Fig. S1*B*) increased to more than twice its resting value during fixed-end force development (red), but this increase was rapidly reversed during unloaded shortening (*SI Appendix*, Tables S1 and S2). *I*_AL1_ at the end of unloaded shortening (green) was not significantly different from that at rest (cyan; *SI Appendix*, Table S2). *I*_AL1_ recovered during force redevelopment after shortening (orange) but then, like *I*_M3_, decreased substantially during isometric relaxation (blue), signaling detachment of a large fraction of the motors that were attached during stimulation at a fixed length. The sixth and seventh actin layer lines, AL6 and AL7, are associated with the right- and left-handed genetic helices that can be traced through the actin monomers (Fig. 4 *C* and *D*, *inset*, respectively). Their intensities (*I*_AL6_; Fig. 4*C*, and *I*_AL7_; Fig. 4*D*) broadly followed the time course of *I*_AL1_ within the resolution of the measurements, and in particular, *I*_AL6_ and *I*_AL7_ decreased during both unloaded shortening and isometric relaxation. Small differences between the time courses of the three actin-based layer lines might be related to their different sensitivities to the working stroke in the actin-attached motors (37).

### The Lattice of Thick and Thin Filaments.

**Fig. 5.**
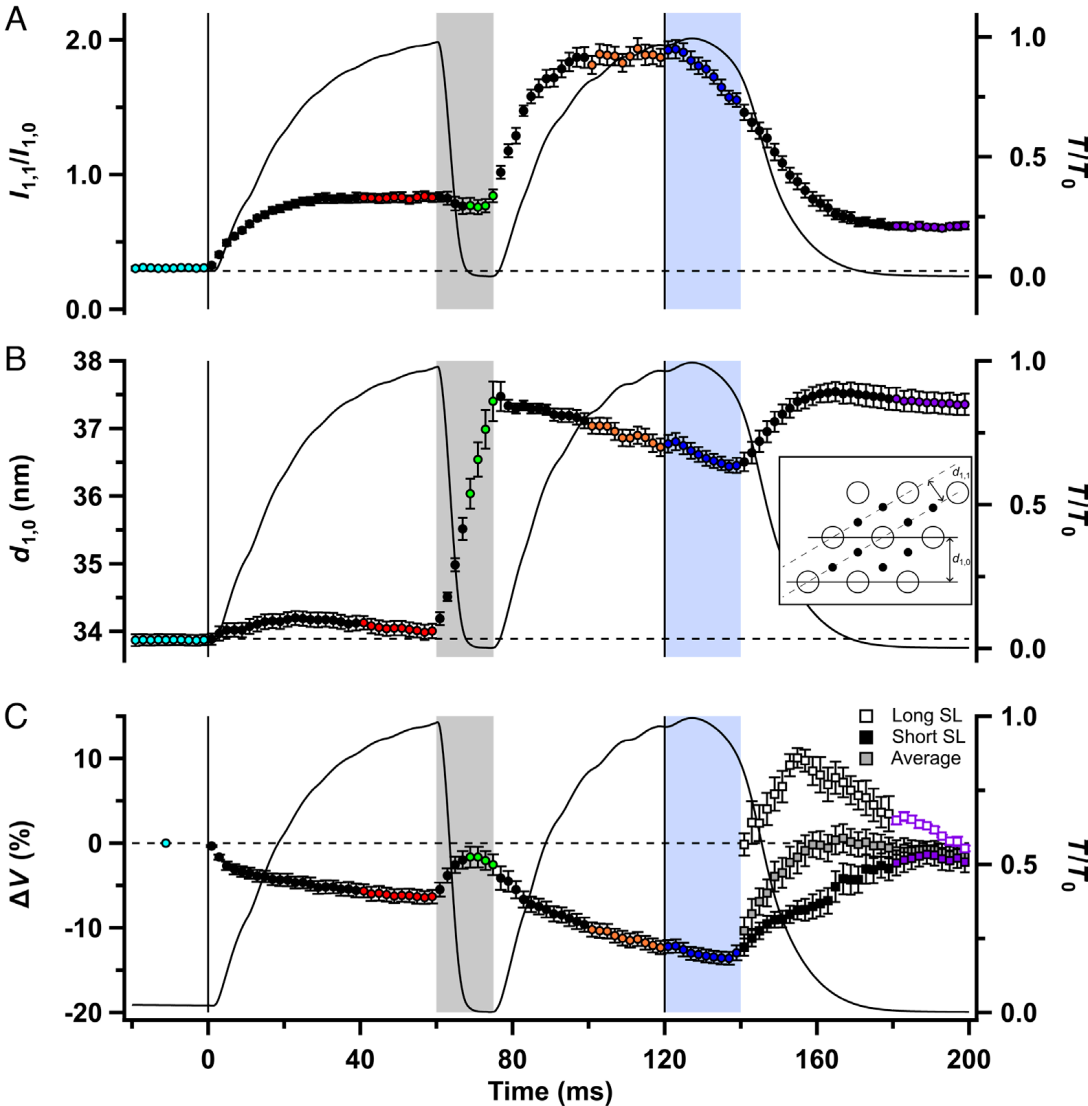
Equatorial X-ray reflections. (*A*) Equatorial intensity ratio (*I*_1,1_/*I*_1,0_). (*B*) Lattice spacing (*d*_1,0_) indicated in the inset as the separation of the 1,0 planes of the filament lattice. (*C*) Volume of the filament lattice (Δ*V*) expressed as a percentage change relative to resting volume. Closed and open squares correspond to Δ*V* calculated from the short and long sarcomere length (SL) populations, respectively. “Average” denotes the average volume of the short and long sarcomere populations. Relative force (*T*/*T*_0_) is shown as the continuous black line. Color coding of the phases of the protocol is shown in Fig. 1*A*. Vertical lines denote the start and end of electrical stimulation; horizontal dashed lines denote the resting values of each parameter. Mean ± SEM for n = 7 muscles in (*A*) and (*B*), and n = 4 muscles in (*C*).

The thick and thin filaments are arranged in a hexagonal lattice (Fig. 5*B*, *Inset*), giving rise to a set of equatorial X-ray reflections (Fig. 1 *C* and *D* and *SI Appendix*, Fig. S1*A*). The ratio of the intensities of the two inner equatorial reflections (*I*_1,1_/*I*_1,0_; Fig. 5*A*) has frequently been used as a signal for the movement of myosin motors from the vicinity of the thick filaments toward the thin filaments (38). Consistent with that interpretation, *I*_1,1_/*I*_1,0_ is low in resting muscle (cyan) when the myosin motors are in the OFF state close to the thick filament surface and increases during fixed-end force development (red) as motors leave the OFF state and bind to the thin filaments. However, *I*_1,1_/*I*_1,0_ is sensitive to factors other than the fractions of OFF and force-generating motors (39), and in the present protocol, it was about three times larger during fixed-end contraction at the shorter SL (Fig. 5*A*, orange) than during the initial contraction at the longer SL (red), despite the similar levels of active force at the two lengths. This difference is much too large to be plausibly explained by the slightly greater overlap between the thick and thin filaments at the shorter SL. It is more likely to be associated with the increase in interfilament separation during shortening, conventionally expressed as the separation between the thick filament planes in the lattice (*d*_1,0_; Fig. 5*B*), which can alter *I*_1,1_/*I*_1,0_ by changing the disorder of filament positions with respect to their ideal hexagonal lattice points. These considerations mean that neither the fraction of myosin motors bound to actin nor their proximity to the thick filament backbone can be determined reliably from measurements of *I*_1,1_/*I*_1,0_ during unloaded shortening. During other phases of the protocol, however, in which the lattice spacing *d*_1,0_ is almost constant, changes in *I*_1,1_/*I*_1,0_ are likely to be dominated by the changes in the fraction of motors close to actin. This would apply, for example, to the large decrease in *I*_1,1_/*I*_1,0_ during isometric relaxation (Fig. 5*A*, blue), in which substantial detachment of motors from actin was previously inferred from the changes in other X-ray reflections described in the preceding section (Fig. 4).

### SL Changes.

**Fig. 6.**
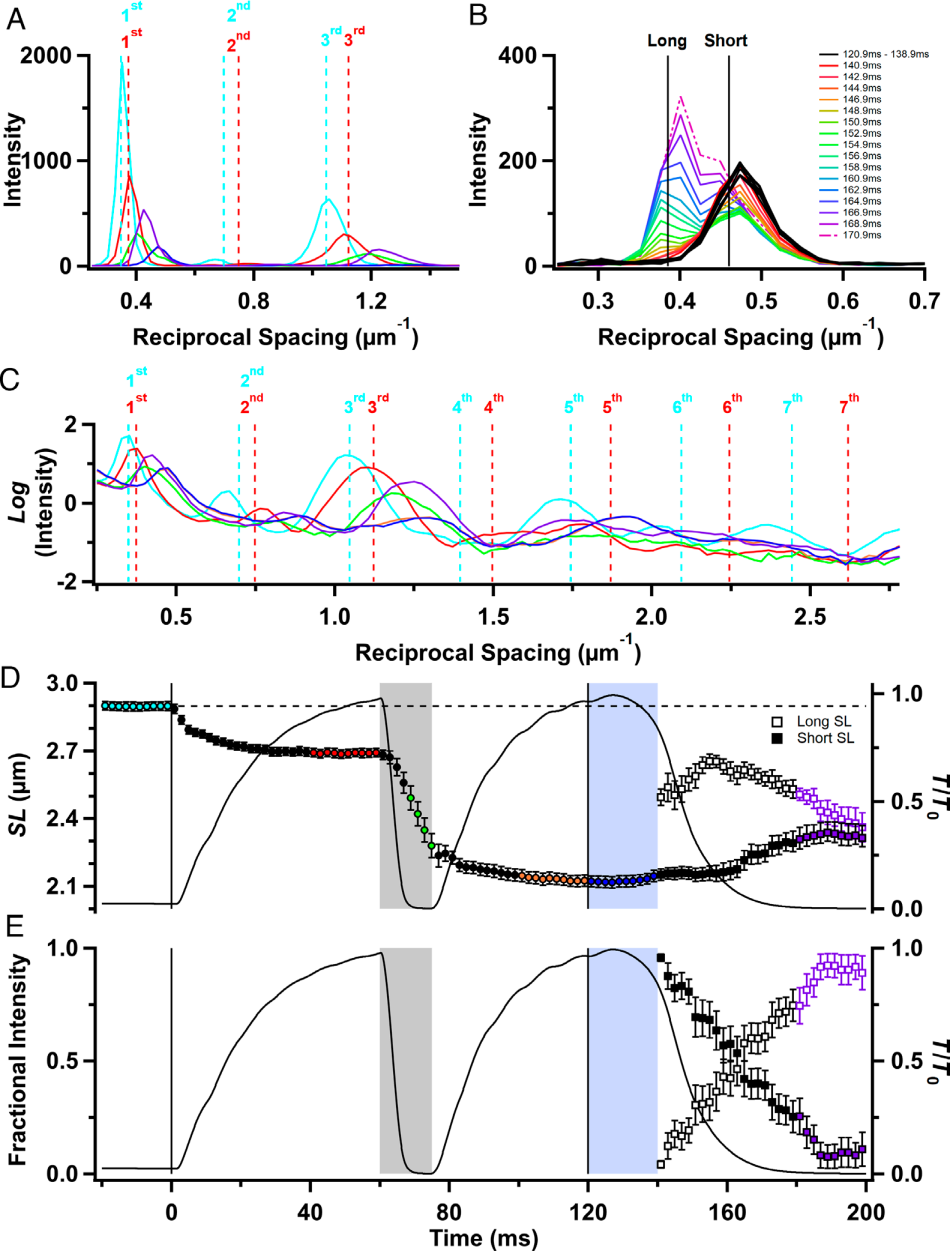
Sarcomere reflections. (*A*) Ultra-low-angle meridional intensity distribution after subtraction of the diffuse background, showing the X-ray reflections corresponding to the first three orders of the sarcomere repeat; colors denote protocol periods shown in Fig. 1*A*. (*B*) First-order sarcomere reflection profiles during isometric relaxation (thicker black), and at the indicated later times. (*C*) Diffracted intensity distribution without removing the diffuse background plotted on a log scale; colors as in (*A*). (*D*) SL is determined from the centroid of the first-order sarcomere reflections. (*E*) Fractional intensities of the first-order reflections from the long and short sarcomere populations. Vertical lines denote the start and end of electrical stimulation. In (*D*) and (*E*), open/closed squares denote long/short sarcomere populations, respectively. Color coding for (*A*) and (*C*) to (*E*) is the same as in Fig. 1*A*. Vertical black lines denote the start and end of electrical stimulation; the horizontal dashed line denotes the resting *SL*. Gray and yellow shaded regions as in Fig. 1*A*. (*A*) to (*C*) show the average of n = 4 muscles; (*D*) and (*E*) are mean ± SEM for n = 4 muscles.

SL changes are fundamental to the protocol used here, so we made time-resolved measurements of SL changes in the same muscles and protocol using ultra-small-angle X-ray diffraction. At least seven orders of sarcomeric X-ray reflections could be recorded with the X-ray detector placed 31 m from the muscle, with the odd orders more prominent (Fig. 6 *A* and *C*). The mean SL was calculated from the centroid of the strong first-order reflection and was 2.90 ± 0.01 µm before stimulation (Fig. 6 *A* and *D*; cyan; *SI Appendix*, Table S2). It decreased to 2.69 ± 0.01 µm (red) during fixed-end force development, as the tendons and more compliant regions at the ends of the muscles were stretched, then almost linearly during unloaded shortening. After force redevelopment, it was 2.13 ± 0.01 µm (orange).

Mean SL was almost constant during isometric relaxation (Fig. 6*D*, blue), confirming the previous results that gave this phase of contraction its name (21, 36, 40, 41). A shoulder in the force record about 20 ms after the last stimulus marks the start of the faster, roughly exponential phase of relaxation, during which force decreased with a rate constant of about 100 s^−1^ (*SI Appendix*, Table S1). Two populations of sarcomeres became detectable at the start of the exponential phase of relaxation (Fig. 6*B*). One population, with SL close to that during isometric relaxation (Fig. 6*D*, closed squares, “Short SL”) was initially dominant but was progressively replaced by a population of longer sarcomeres (Fig. 6*D*, open squares, “Long SL”). By about 40 ms after the last stimulus, when force had fallen to about 10% of its peak value, the intensities of the long and short sarcomere reflections were roughly equal (Fig. 6*E*). During the next 40 ms, the SLs of the two populations converged, so that only a single population was detectable (Fig. 6 *A* and *C*–*E*; purple).

By analogy with previous measurements on short segments of isolated muscle fibers (36, 40) and myofibrils (41), the short and long populations of sarcomeres observed during exponential relaxation may be associated with transiently stronger and weaker regions of the muscle fibers, most likely resulting from regional variation in the rate of filament inactivation. Unexpectedly, the amplitude and kinetics of these SL changes were reproducible between and along muscles, suggesting that this dynamic instability of sarcomere populations is not “chaotic” in the usual sense of the word.

Finally, because SL (Fig. 6*D*) and filament lattice spacing (Fig. 5*B*) were measured in the same regions of the muscles, we were able to calculate changes in the lattice volume (Δ*V*; Fig. 5*C*) at each time point during the protocol. Δ*V* decreased by about 8% during fixed-end force development, partly recovered during unloaded ramp shortening, then decreased again by about 10% during force redevelopment at the shorter length. These changes are larger than those reported previously in isolated muscle fibers from amphibians (42). Mouse EDL muscles do not exhibit constant volume behavior during fixed-end contraction; the filament lattice is compressed by force-generating myosin motors. This compression is maintained and even slightly increases during isometric relaxation. The presence of two sarcomere populations during exponential relaxation leads to two estimates of lattice volume (Fig. 5*C*). It was not possible to resolve the *d*_10_ values for the two populations (*SI Appendix*, Fig. S1*A*), but the mean of the two lattice volume estimates (Fig. 5*C*, gray squares) recovered its resting value even before mechanical relaxation was complete, signaling an increase in lattice volume of almost 15% in less than 20 ms, presumably associated with the restoration of the resting volume as the lattice compression by force-generating myosin motors is removed.

## Discussion

### Muscle Activation at the Start of Electrical Stimulation.

**Fig. 7.**
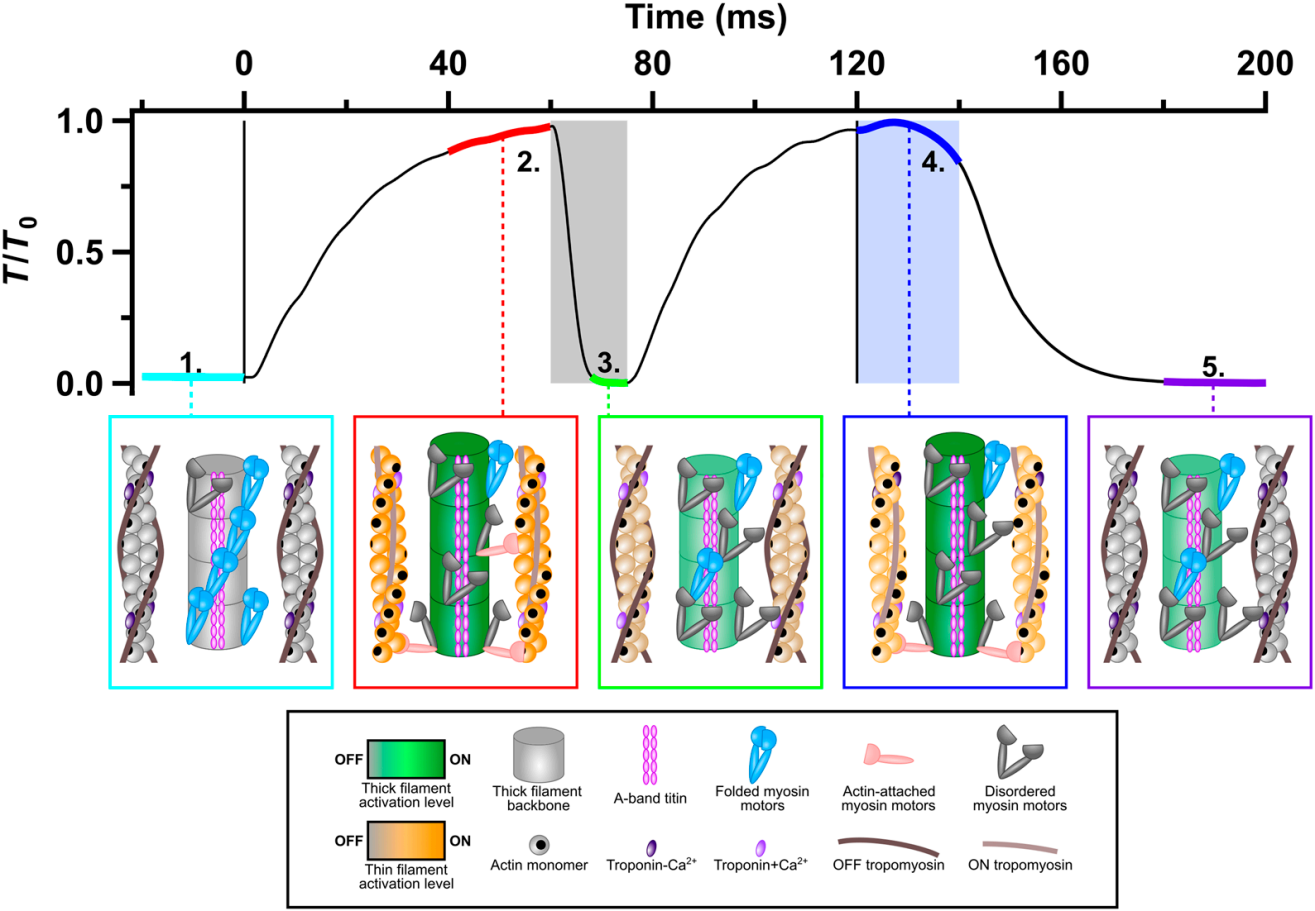
Thick and thin filament activation states and myosin motor conformations. *Upper* panel: Force normalized to maximal force (*T*/*T*_0_). Vertical black lines denote the start and end of electrical stimulation. *Lower* panels: Schematic of thin and thick filaments and myosin motor conformations at the five periods marked on the force trace. The filament components are identified in the key box. The activation state of the thick filament is coded as gray (OFF) to green (ON); that of the thin filament as gray (OFF) to orange (ON).

The aim of the experiments described above was to identify how changes in intracellular free calcium concentration [Ca^2+^]_i_ and muscle load control the regulatory states of the thin and thick filaments and thereby provide a mechanistic explanation of the physiological kinetics of contraction and relaxation in intact mammalian muscle. In resting muscle (Fig. 7, box 1, cyan), [Ca^2+^]_i_ is low, and both the thin and thick filaments are OFF (gray). Most myosin motors (blue) are folded back against their tails in helical tracks on the surface of the thick filament, although there are some disordered motors (dark gray). Tropomyosin (dark brown) covers the myosin binding sites on actin (black). After 40 to 50 ms of stimulation at the initial muscle length (Fig. 7, Box 2, red), calcium has bound to troponin (light purple), and tropomyosin has moved to the center of the groove between the strands of the actin helix (light brown); the thin filament is ON (orange). Most myosin motors have left the helical tracks characteristic of the OFF state; the thick filament is ON (green), and some motors (light red) are bound to actin and generating force. Both filaments are axially strained compared with the resting state.

The transitions between the resting (cyan) and fixed-end contracting (red) states occur at different speeds in the different structures. [Ca^2+^]_i_ increases very rapidly following electrical stimulation, and the thin filaments become calcium-saturated with a half-time of less than 2 ms (18, 19) (Fig. 1*A*), but force develops more slowly, with a half-time of about 17 ms (*SI Appendix*, Table S1). The time course of attachment of myosin motors to actin (*I*_AL1_; Fig. 4*B* and *SI Appendix*, Table S1) (37), follows that of force, but the thin filaments (*I*_AL2_ and *S*_A13_; Fig. 3 and *SI Appendix*, Table S1) and thick filaments (*A*_ML1_, *A*_ML4_, *S*_M6_, and *S*_M11_; Fig. 2 and *SI Appendix*, Table S1) are both activated much faster, with half-times of about 6 ms. These results show that thin and thick filament activation at the start of electrical stimulation in fixed-end conditions is slower than the rise of [Ca^2+^]_i_ but faster than that of myosin binding to the thin filaments, active force, and filament load.

As far as the thin filaments are concerned, this conclusion is qualified by the relatively low signal-to-noise ratio of the relevant structural signals (Fig. 3), and the present data do not exclude the possibility that the time course of thin filament activation is the sum of a fast [Ca^2+^]_i_-related component and a slower component that follows myosin binding and force production, as suggested previously (16, 25, 43). Such a biphasic time course would correspond to sequential transitions between the three regulatory states of the thin filament deduced from biochemical and structural studies on isolated proteins, called blocked, closed, and open (44), with the blocked-to-closed transition gated by calcium binding and the closed-to-open transition by myosin binding.

The time course of thick filament activation is determined with a much higher signal-to-noise ratio by the relevant structural signals (Fig. 2 and *SI Appendix*, Table S1), showing that any temporal components synchronous with either [Ca^2+^]_i_ or muscle load are small. The absence of a direct [Ca^2+^]_i_-dependent component of thick filament activation has been demonstrated more directly by imposing zero-load shortening at the start of stimulation (11, 14), showing that thick filaments remain OFF when [Ca^2+^]_i_ increases at zero load. However, if thick filament activation is solely determined by the load, the difference in time courses shows that the relationship is highly nonlinear or that there is a slow kinetic step between the load change and the change in filament activation. Additional experiments will be required to distinguish these possibilities.

### Load Dependence of Filament Inactivation.

We determined the effect of removing the load at full calcium activation on the activation states of the thick and thin filaments by imposing rapid shortening at the plateau of a tetanic contraction. Both the thin and thick filaments switch OFF very rapidly when the load is removed in these conditions so that by the end of the shortening period (Fig. 7, box 3, green), the activation state of the thin filaments (orange-gray) has returned almost to the resting level (Fig. 3*A* and *SI Appendix*, Table S2), although calcium is still bound to troponin (light purple). The thick filaments (light green) are about half ON (Fig. 2*A* and *SI Appendix*, Table S2). Very few actin-bound myosin motors are required to drive unloaded shortening (35). The axial periodicities of the thin and thick filaments return to close to their resting levels.

The X-ray signals associated with thick filament activation (*I*_ML1_, *I*_ML4_, *S*_M6_, and *S*_M11_; Fig. 2) and thin filament activation (*I*_AL2_ and S_A13_; Fig. 3) decreased with half-times of about 4 ms (*SI Appendix*, Table S1) in this protocol, exactly matching that of the detachment of myosin motors from actin (*I*_AL1_, *I*_AL6_, and *I*_AL7_; Fig. 4). Within the resolution of the data, all these changes are simultaneous with the decrease in load. The intensity of the M3 reflection from the axial repeat of the myosin motors (*I*_M3_; Fig. 4*A*) decreased even faster, which is expected because it is sensitive to the working stroke in the actin-bound motors before they detach (33).

We conclude that high load or the presence of actin-bound force-generating myosin motors are required for the fully ON state of both the thin and thick filaments and that both filaments switch partly OFF within a few milliseconds when that condition is removed, despite maintained saturating [Ca^2+^]_i_. The conclusion that actin-bound force-generating myosin motors are necessary for the fully activated state of the thin filament is consistent with the results of a recent study that used fluorescent probes on troponin to monitor its activation state in demembranated fibers from mammalian muscle (16). In contrast, a previous SAXD study on demembranated muscle fibers reported no change in *I*_AL2_ when the load was decreased to about one-third of the isometric value (45), suggesting that an intermediate load may be sufficient to maintain the fully activated state of the thin filament.

In the case of the thick filaments, the activation state attained after removal of the load and signaled by the structural parameters *I*_ML1_ (Fig. 2*A*) and *S*_M6_ (Fig. 2*C*) is clearly distinct from the fully OFF state of the thick filament observed in resting muscle, but matches the intermediate activation state observed after mechanical relaxation at the end of electrical stimulation (Fig. 7; box 5, purple), when [Ca^2+^]_i_ is low, no Ca^2+^ is bound to troponin, and the thin filament is OFF (gray). These results show that the thick filament also has three regulatory states and that its inactivation is not solely controlled by the load, but has an intrinsically slow load-independent component that may be related to the phenomenon of posttetanic potentiation (21). Mechanical relaxation does not require the fully OFF state of the thick filament. Intriguingly, the spacing of the 11^th^ myosin-based meridional reflection (*S*_M11_; Fig. 2*D*), associated with the domain repeat of titin in the thick filament backbone (9, 10) (Fig. 7, magenta), did recover fully during mechanical relaxation, suggesting that it may report the fast load-dependent component.

### Calcium Dependence of Filament Inactivation.

We used the period following the end of electrical stimulation (Fig. 7, box 4; blue) to determine how the filaments inactivate when [Ca^2+^]_i_ decreases at high load, i.e. the converse experiment to decreasing the load at saturating [Ca^2+^]_i_ described above. At the end of electrical stimulation, [Ca^2+^]_i_ decreases at about 130 s^−1^ (18) (Fig. 1*A*), but the load remains high for about 20 ms in the period called isometric relaxation in which sarcomeres are isometric (Fig. 6*B*, black; Fig. 6*D*, blue). The X-ray signals of the fraction of myosin motors attached to actin (*I*_AL1_, *I*_AL6_, *I*_AL7_, and *I*_M3_; Fig. 4) indicate that the majority of myosin motors detach from actin during this period, at a rate of about 40 s^−1^ (*SI Appendix*, Table S1), implying that the force per attached motor is increasing, as observed in single fibers of amphibian muscle at 4 °C (36). All the structural signals related to thick filament activation (Fig. 2) indicate that thick filaments stay fully ON (Fig. 7, box 4; green thick filament) when [Ca^2+^]_i_ decreases during isometric relaxation, as expected from the hypothesis that their activation state is strongly dependent on load but independent of [Ca^2+^]_i_. These results, therefore, reinforce the conclusions from the period in which the load was removed at full calcium activation and discussed above, as well as those of previous studies in which the load was held low at the start of stimulation (11, 14).

Throughout the above discussion, for simplicity, we have focused on the dependence of filament activation on the load, following the original hypothesis of thick filament mechanosensing (11). The recovery of the X-ray parameters associated with filament activation following low-load shortening make it unlikely that the observed changes in these parameters are related to changes in SL per se, filament lattice spacing, or the history-dependent properties of muscle contraction. However, given the reciprocal relationship between load and shortening velocity in muscle, in all the protocols described above, high load and force-generating actin-bound myosin motors are correlated with isometric conditions, and low load and few actin-bound motors with rapid shortening. Therefore, it remains possible that the effects of low load and few actin-bound motors are, in fact, due to rapid filament sliding, and those of high load and many actin-bound motors are a property of the isometric or near-isometric state. Such an effect might be mediated by myosin binding protein-C (MyBP-C), the N terminus of which can either bind to and activate the thin filaments (46–48) or bind to myosin and stabilize the OFF state of the thick filaments (49). In the conditions of the present experiments, filaments slide at about 10 nm per millisecond during unloaded shortening, so the N termini of any MyBP-C molecules that are bound to actin must detach on the millisecond timescale, suggesting a plausible mechanism for the decrease in the levels of activation of both the thin and thick filaments.

Independent of the mechanism, the present results show that the thick filaments remain fully activated as [Ca^2+^]_i_ decreases during isometric relaxation (Fig. 7, box 4, blue). The thin filaments also remain almost fully on, as signaled by the mean value of *I*_AL2_ during this period being about 80% of that at peak force at the same length (Fig. 3*A* and *SI Appendix*, Table S2 and Fig. 7, box 4, orange thin filament). This result suggests that the myosin motors that remain attached to actin in this period are sufficient to maintain the activated state of the thin filament as determined by *I*_AL2_ despite the reduction in [Ca^2+^]_i_, an effect that may be enhanced by the higher force per attached motor (50). However, this conclusion raises the more fundamental question of why there are fewer actin-attached myosin motors during isometric relaxation if the thin filaments remain almost fully activated, as assessed by *I*_AL2_. This result suggests that the feature of thin filament structure that controls the attachment rate is distinct from the azimuthal position of tropomyosin reported by *I*_AL2_, which remains in the fully activated or open position. Recent high-resolution cryo-EM structures of the thin filament (6, 51, 52) support such a distinction, indicating that troponin, as well as tropomyosin, can block the binding of myosin motors to actin. Although tropomyosin stays almost completely in the blocking position during isometric relaxation, we infer from the decreased fraction of motors attached to actin that troponin more closely follows [Ca^2+^]_i_ and moves back to its inhibitory conformation, although direct structural evidence for such movement is lacking.

### The Mechanism of Muscle Relaxation.

Finally, we return to the fundamental question posed in the Introduction: How does muscle relax following electrical stimulation when the load is high, activating the thick filament, and myosin motors are bound to actin, preventing the return of tropomyosin to its OFF conformation (Fig. 7, box 4)? The present results suggest the following sequence of events. Initially, sarcomeres remain isometric (Fig. 6*D*), and force remains high, although the rate of attachment of new motors to actin decreases, probably mediated by structural changes in troponin following the decrease in [Ca^2+^]_i_ as discussed above. The strain in the remaining actin-attached motors increases, and at about 10 ms after the end of stimulation force starts to decline, leading to sarcomere stretch as the tendons shorten. At about 20 ms, some sarcomeres start to yield, and the distribution of SLs becomes bimodal. Both populations of sarcomeres are then stretched further as the force decline accelerates, leading to more tendon shortening, and subsequently becomes exponential, with a rate constant of about 100 s^−1^. By less than 10 ms after the end of isometric relaxation, the fraction of actin-attached motors (*I*_AL1_; Fig. 4*B*; *I*_M3_; Fig. 4*A*) has already reached the steady value characteristic of full mechanical relaxation. The lengths of the two sarcomere populations then start to converge, although this process is not complete until about 50 ms later. All these events are much faster than the final recovery of the folded helical OFF state of the thick filament (*I*_ML1_, *I*_ML4_; Fig. 2*A* and *B*; *I*_M3_; Fig. 4*A*), which requires much longer than the 80-ms recording period following the last stimulus.

Phenomenologically, these two phases of relaxation have been described previously in isolated muscle fibers (36, 40) and myofibrils (41, 53) as a slow, almost linear sarcomere-isometric phase followed by a fast, almost exponential phase in which some sarcomeres shorten at the expense of others being stretched. Those previous experiments determined SL changes during relaxation by visible light imaging or diffraction, which can be influenced by the selection of local sarcomere populations by Bragg angle effects (54). Moreover, SLs cannot be determined reliably in whole muscles like mouse EDL by visible light diffraction because the light is diffracted many times as it traverses the thickness of the muscle. These limitations are removed by ultra-low-angle X-ray diffraction, in which the X-ray photons are diffracted only once, providing a precise measurement of the SL distribution for all the myofibrils in the X-ray beam. In the fast exponential phase of muscle relaxation, the same two populations of SLs were observed at each time in different places along each muscle and in different muscles. This remarkable reproducibility contrasts with previous studies on isolated myofibrils and single muscle fibers, suggesting that it is intrinsic to the distribution of sarcomeres along and across the muscles, acting together with the tendon compliances (55). Those systematic properties of the muscle–tendon complex determine the physiological time course of relaxation.

## Materials and Methods

Full details of the *Materials and Methods* are provided in *SI Appendix*.

### Muscle Preparation.

Intact mouse EDL muscles were dissected from the hindlimb of male C57BL/6 J mice aged 4 to 8 wk as described previously (14, 21). Muscles were mounted vertically in a custom 3D printed trough at beamline ID02 of the ESRF (Grenoble, France) (56), attached to a dual-mode force/length transducer (300C-LR, Aurora Scientific, Aurora, Canada) and continuously perfused with Krebs–Henseleit solution equilibrated with carbogen at 28 °C. Electrical stimuli were provided by a high-power biphasic stimulator (701C, Aurora Scientific) via parallel platinum electrodes. The stimulus voltage was 1.5 times the required amount to elicit the maximum twitch force response, and optimal muscle length (*L*_0_) was set to produce maximum force in response to a 100-ms train of stimuli at 110 Hz.

### SAXD Data Collection.

SAXD patterns were recorded using an Eiger 2 4 M detector (Dectris Inc., Baden, Switzerland) and normalized for the detector response, incident intensity, and diffraction geometry (56). The sample-to-detector distance was initially set to 31 m to measure SL, then to either 3.2 or 2.0 m to record X-ray reflections associated with filament periodicities in the 60 - 3.5 nm range.

Muscles were aligned in the X-ray beam using a sample-to-detector distance of 31 m using 1 ms exposures with a 50 μm rhodium attenuator with 3% transmission. Following alignment, the rhodium attenuator was replaced with a 20 μm lead attenuator with 21% transmission. The stimulus and length change protocol is shown in Fig. 1*A*. X-ray diffraction data were acquired in 110 time frames, each with 1.8 ms integration and 0.2 ms latency time. To minimize radiation damage, X-ray exposure was limited by a fast shutter, and muscles were moved vertically and horizontally between successive X-ray exposures. X-ray data were averaged from 15 to 35 contractions per muscle. Records in which force had declined more than 15% from the first stimulus were excluded from further analysis.

Force, stimulus, muscle length, and X-ray acquisition timing were sampled and analyzed using custom-made software written in LabVIEW (National Instruments).

### SAXD Data Analysis.

Normalized data were analyzed using SAXSutilities2, SAXS package (P. Boesecke, ESRF, Grenoble, France), Fit2D (A. Hammersley, ESRF, Grenoble, France), ImageJ [National Institute of Health, Bethesda, MD (57)] and Igor Pro 8 (WaveMetrics, Inc., Portland, OR). SAXD patterns containing collagen-based reflections (~2 to 6 per muscle), indicating the presence of tendons in the X-ray beam, were excluded from the analysis.

The instrument background was subtracted from each normalized pattern, and useable records from the same muscle were averaged. Patterns were corrected for any tilting of the muscle with respect to the detector pixels using a custom-written ImageJ macro. Processed images were integrated to produce axial and radial intensity distributions of the meridional, layer line, and equatorial reflections (*SI Appendix*, Fig. S1). Background intensity was subtracted and intensities and spacings of X-ray reflections were determined by Gaussian fitting or integrations.

## Supplementary Material

Appendix 01 (PDF)

Dataset S01 (XLSX)

## Data Availability

Source data have been deposited in Figshare (https://doi.org/10.6084/m9.figshare.28400867) (58). All study data are included in the article and/or supporting information.
