# Supplementary material for: Dual-filament regulation of relaxation in mammalian fast skeletal muscle
Source: Proc Natl Acad Sci U S A. 2025 Mar 12;122(11):e2416324122. doi: 10.1073/pnas.2416324122 (PMC11929500; doi:10.1073/pnas.2416324122)
Supplement: Supplementary file 1 — Appendix 01 (PDF) [file pnas.2416324122.sapp.pdf]

PNAS

Supporting Information for

Dual Filament Regulation of Relaxation in Mammalian Fast Skeletal Muscle

Cameron Hill, Michaeljohn Kalakoutis, Alice Arcidiacono, Flair Paradine Cullup, Yanhong Wang, Atsuki Fukutani, Theyencheri Narayanan, Elisabetta Brunello, Luca Fusi, Malcolm Irving

Cameron Hill
Email: cameron.hill@kcl.ac.uk

This PDF file includes:

- Supporting text
- Figure S1
- Tables S1 to S3
- Legend for Dataset S1
- SI References

Other supporting materials for this manuscript include the following:

- Datasets S1

Supporting Information Text

Materials and Methods

Animals and Muscle Preparation

Male mice (strain C57BL/6J) aged 4-8 weeks were housed at the European Synchrotron Radiation Facility (ESRF) Biomedical Facility, Grenoble, France, in 12:12 hour light:dark cycles at 50% relative humidity, with ad libitum access to water and a standard lab diet. Animals were sacrificed via cervical dislocation, followed by a confirmation method of permanent cessation of circulation by severing the femoral artery, in compliance with the UK Home Office Animals (Scientific Procedures) Act 1986, Schedule 1 and European Union regulation (directive 2010/63). After sacrifice, whole extensor digitorum longus (EDL) muscles were dissected from the hindlimb under a stereomicroscope in a trough continuously perfused with Krebs-Henseleit solution (composition in mM: NaCl 118; KCl 4.96; $MgSO_4$ 1.18; $NaHCO_3$ 25; KH_2PO_4 1.17; glucose 11.1; $CaCl_2$ 2.52) with a pH ~7.4 at room temperature after equilibration with carbogen (95% O_2, 5% CO_2). Metal hooks were tied with 5-0 silk sutures at the proximal and distal tendons of the muscle to allow attachment to the experimental setup. The muscle was mounted in a custom 3D-printed resin chamber between a fixed hook and the lever of a dual-mode force/length transducer (300C-LR, Aurora Scientific, Aurora, Canada) and continuously perfused with Krebs-Henseleit solution equilibrated with carbogen at 27-28 ˚C.

Electrical stimuli were provided by a high-power biphasic stimulator (701C, Aurora Scientific) via parallel platinum electrodes. The muscle was placed between a fixed mylar window and a second window attached to a 3D-printed screw to allow it to be positioned as close as possible to the muscle to minimise the X-ray path in the solution. The stimulus voltage was 1.5 times the required amount to elicit the maximum twitch force response. Optimal muscle length (L_0) was set to produce maximum force in response to a 100-ms train of stimuli at 110 Hz repeated at 5-minute intervals. L_0 was 13.4 ± 0.1 mm. Muscle cross-sectional area was estimated as (2 x (W_{MW}))/(ρ*L_0), where ρ = 1.06 $g.cm^{-3}$ is the density of the muscle, and W_{MW} is the muscle wet weight. W_{MW} was 10.6 ± 0.6 mg, giving a cross-sectional area of 1.46 ± 0.08 mm^2. Plateau force in fixed-end tetani at L_0 was 270.6 ± 21.8 kPa (mean ± SEM; n=7).

Small-angle X-ray Diffraction Data Collection

The trough was sealed to prevent solution leakage, and the muscle was mounted vertically at L_0 at beamline ID02 of the ESRF (Grenoble, France) (1) to take advantage of the smaller vertical beam focus to optimise spatial resolution along the meridional axis. The monochromatic X-ray beam provided 6 x 10^{12} $photons.s^{-1}$ at 0.1 nm wavelength with full-width at half maximum about 140 μm horizontally and 40 μm vertically at the sample (2). Small-angle X-ray diffraction (SAXD) patterns were recorded using an Eiger 2 4M detector (Dectris Inc., Baden, Switzerland) of active area 155.1 mm x 162.15 mm, with 2068 x 2162 pixels, each 75 μm x 75 μm, organised as 2 x 4 modules (HxV) with small gaps between modules. The sample-to-detector distance was initially set to 31 m to measure sarcomere length, then to either 3.2 or 2.0 m to record X-ray reflections associated with filament periodicities in 60 - 3.5 nm range. The detector position in each case was optimised so that X-ray reflections of interest did not fall in the gaps between detector tiles. The 3.2 m sample-to-detector distance was used to measure changes in the equatorial reflections (1,1 and 1,0), the third-order meridional reflection (M3), the sixth-order meridional reflection (M6), the mixed first-order actin layer line (AL1) and myosin layer line (ML1), the fourth-order myosin layer line (ML4) and the sixth- and seventh-order actin layer lines (AL6 and AL7 respectively). The 2.0 m sample-to-detector distance was used to measure changes in the equatorials, ML1, AL1, the second-order actin layer line (AL2) and the eleventh-order meridional reflection (M11).

Muscles were aligned in the X-ray beam using a sample-to-detector distance of 31 m and 1 ms exposures with a 50 μm rhodium attenuator with 3% transmission. Rapid assessment of two-dimensional X-ray patterns was provided by SAXSutilities2 (3). Following alignment, the rhodium

attenuator was replaced with a 20 µm lead attenuator with 21% transmission. The main features of the stimulus and length change protocol are shown in Fig. 1*A*. X-ray diffraction data were acquired in 110 time frames, each with 1.8 ms integration and 0.2 ms latency time. To minimise radiation damage, X-ray exposure was limited by a fast shutter, and the muscle was moved vertically and horizontally between successive X-ray exposures. X-ray data were added from 15 to 35 contractions per muscle. Records where force had declined more than 15% from the first record were excluded from further analyses.

Force, stimulus, muscle length, and X-ray acquisition timing were sampled and analysed using custom-made software written in LabVIEW (National Instruments).

SAXD Data Analysis

SAXD patterns were analysed using SAXSutilities2, SAXS package (P. Boesecke, ESRF, Grenoble, France), Fit2D (A. Hammersley, ESRF, Grenoble, France), ImageJ (National Institute of Health, Bethesda, USA (4)) and Igor Pro 8 (WaveMetrics, Inc., Portland, USA). SAXD patterns (~2-6 per muscle) containing collagen-based reflections, indicating the presence of tendons in the X-ray beam, were excluded from the analysis.

Analysis of Ultra-Small-Angle X-Ray Diffraction Patterns

The sarcomeric X-ray reflections were recorded with a sample-to-detector distance of 31 m at three places along the long axis of each muscle. Diffraction data were integrated 0.56 μm^{-1} on either side of the meridional axis, and the residual background intensity was removed using an arc hull algorithm using the Baselines extension in Igor Pro. The axial region 0.25-0.57 μm^{-1} containing the first-order sarcomere reflections was fitted with a single Gaussian function for time frames up to 140 ms (Fig. 6*D*). At later times during relaxation, two peaks could be resolved in this region, which were fitted with two Gaussians with different axial widths (Fig. 6*B*,*D*).

Analysis of SAXD Patterns

The instrumental background was subtracted from the SAXD data for each time frame averaged from the series of contractions in each muscle, and each resulting image was centred and aligned using the equatorial 1,0 reflections.

Equatorial Reflections

The equatorial intensity distribution was determined by integrating from 0.0036 nm^{-1} on either side of the equatorial axis (perpendicular to the muscle axis), and the intensities and spacings of the 1,0, Z-disc, 1,1 and 2,0 reflections were determined by fitting four Gaussian peaks in the radial region 0.02 to 0.065 nm^{-1} with the following constraints:

$$d_{1,1} = \frac{d_{1,0}}{3^{\frac{1}{2}}}$$

$$d_{2,0} = \frac{d_{1,0}}{2}$$

$$d_z = d_{1,0} \times \text{constant}$$

The volume of the filament lattice in each sarcomere (*V*) was calculated as:

$$V = d_{1,0}^{\ 2} \times \frac{2}{\sqrt{3}} \times \text{Sarcomere Length}$$

Meridional Reflections

To analyse the meridional, equatorial and layer line reflections, aligned SAXD patterns were mirrored horizontally and vertically. The distribution of diffracted intensities along the meridional axis of the diffraction pattern (parallel to the muscle axis) was calculated by integrating from 0.015 nm^{-1} on either side of the meridian. For the meridional and equatorial reflections, intensity distributions were background subtracted using polynomial fitting with the Baselines extension in Igor Pro. Integrated intensities were obtained from the following axial regions: M3, 0.066–0.072 nm^{-1}; M6, 0.135–0.142 nm^{-1}; M11, 0.246-0.260 nm^{-1}. The cross-meridional width of the M3 and M6 reflection was determined from the radial intensity distribution in the axial region defined above using a single Gaussian centred on the meridian. The interference components of the M3 and M6 reflections were characterised by fitting multiple Gaussian peaks with the same axial width to the meridional intensity distribution. For the M11 reflection, 1:2:1 smoothing of the integrated intensity as a function of time was performed to increase the signal-to-noise ratio. It was then fitted with a single Gaussian. The total intensities of the M3 and M6 reflections were calculated as the sum of the intensities of the component peaks of each reflection and multiplied by the cross-meridional width to correct for changes in lateral misalignment between filaments (5); the spacing was calculated as the weighted average of that of the component peaks. In some time frames, an additional reflection called the 'star' peak (6–8) was observed on the low-angle side of the M3 reflection. This peak was not considered to be a component of the M3 reflection (6–8).

Layer Line Reflections

The intensities of the first myosin and first actin layer lines (ML1 and AL1) were calculated by integrating the radial region between 0.037 nm^{-1} and 0.064 nm^{-1} from the meridional axis for 3.2 m and 2 m data. Due to the partial overlap of ML1 and AL1, integrated intensities were obtained from the axial region 0.017-0.033 nm^{-1} for each muscle and separated by global Gaussian deconvolution of the time series data under the simplifying assumption that their spacings, S_{AL1} and S_{ML1} respectively, and axial widths do not change during contraction. An independent estimate of S_{AL1} (Table S2) was obtained from the spacings of the AL6 and AL7 layer lines (S_{AL6} and S_{AL7} respectively) using the equation (9):

$$S_{AL1} = \frac{1}{\left(\left(\frac{1}{S_{AL7}}\right) - \left(\frac{1}{S_{AL6}}\right)\right)}$$

The intensity of the ML4 layer line was obtained by integrating radially in the reciprocal range 0.030-0.077 nm^{-1} parallel to the meridian (10). Due to the proximity of the ML4 reflection to a detector tile boundary in unmirrored data, this integration was performed on one half of SAXD patterns. Furthermore, an additional reflection, the meridional AM_{+1} reflection at ~0.10 nm^{-1}, partially overlaps the ML4 reflection on the high-angle side (Fig. S1*C*) (10). The intensity of the ML4 reflection was therefore obtained by integrating the lower angle region of the reflection in the axial range 0.088-0.093 nm^{-1} (Fig. S1*C*).

The intensity of the AL2 layer line was obtained by integrating the radial region 0.160-0.303 nm^{-1}, then integrating axially in the region 0.0373-0.0729 nm^{-1} (11, 12). The second actin layer line is not detectable in resting muscle (13), so changes in its integrated intensity were normalised to the value for peak force long (Fig. 3a, red).

Intensity distribution profiles for the AL6 and AL7 reflections were obtained by integrating in the radial region 0.035-0.060 nm^{-1} (9), with the lower limit chosen to minimise the contribution of the tails of the M7, M8 and M9 reflections (Supplementary Fig. 1*D*,I). Due to the proximity of the AL6 reflection to a detector tile boundary, this integration was performed on one half of the SAXD pattern. The integration for the weaker AL7 reflection was performed on fully mirrored SAXD patterns to maximise signal-to-noise ratio. The spacings and intensities of the AL6 and AL7 reflections were obtained by global Gaussian deconvolution of the axial regions between 0.166-0.178 nm^{-1} for AL6

and 0.191-0.215 nm^{-1} for AL7, respectively (14), assuming that the spacings of the M7 and M8 reflections and the axial width of all the reflections did not change during contraction.

The thirteenth-order actin-based meridional reflection (A13), which corresponds to the fundamental ~2.7 nm axial repeat of actin monomers in the thin filament, was outside the detector in the present experiments, but its spacing was calculated from S_{AL6} and S_{AL7} using the equation (9):

$$S_{A13} = \frac{1}{\left(\frac{1}{S_{AL6}}\right) + \left(\frac{1}{S_{AL7}}\right)}$$

For all actin and myosin layer line reflections, the axially integrated 1D profiles were firstly 1:2:1 smoothed as a function of time to increase the signal-to-noise ratio of the weakly diffracting reflections. For the ML1/AL1 layer line axial integrations, the background was removed by selecting regions not containing higher order layer line reflections and the intensity distribution of the background was fitted with a double Gaussian and then subtracted using the Baselines extension. The residual background intensity was removed by polynomial fitting and subtracted for the remaining layer line reflections.

Correction of Integrated Intensities for changes in muscle mass in the X-ray beam

Small movements of the muscles with respect to the X-ray beam during contraction can alter the scattering mass in the beam and, consequently, the diffracted X-ray intensities. All reported X-ray intensity time-courses were corrected for this effect by normalising with respect to the background under the ML1 layer line at each frame.

Statistical Analyses

All statistical analyses were performed using Jamovi (The Jamovi Project (15), v2.5.6) and Microsoft Excel.

Paired-sample T-tests were used to determine whether the half-time and rate constants for X-ray data differed significantly from that of force (Table S1). When data were not normally distributed, as determined by checks for normality of distribution by Shapiro-Wilks test, skewness, and kurtosis, the non-parametric Wilcoxon's signed-rank test was used.

Differences in force or X-ray data between the key time periods in the protocol shown in Fig. 1 were analysed using a repeated measures analysis of variance (ANOVA) with Tukey's post hoc analysis for data where a main effect was observed (Table S2). To determine whether non-parametric analyses were required, data were first checked for normality of distribution using Shapiro-Wilks test, skewness, kurtosis and sphericity using Mauchley's W. Those which were not spherical used a Greenhouse-Geisser sphericity correction. In the event of non-normally distributed data, the non-parametric Friedman's test with Durbin-Conover pairwise comparisons was used. Main effects and post-hoc analyses P-values are provided in Table S3.

Data presented are mean ± S.E.M. throughout. Significance was set at P<0.05 for all analyses.

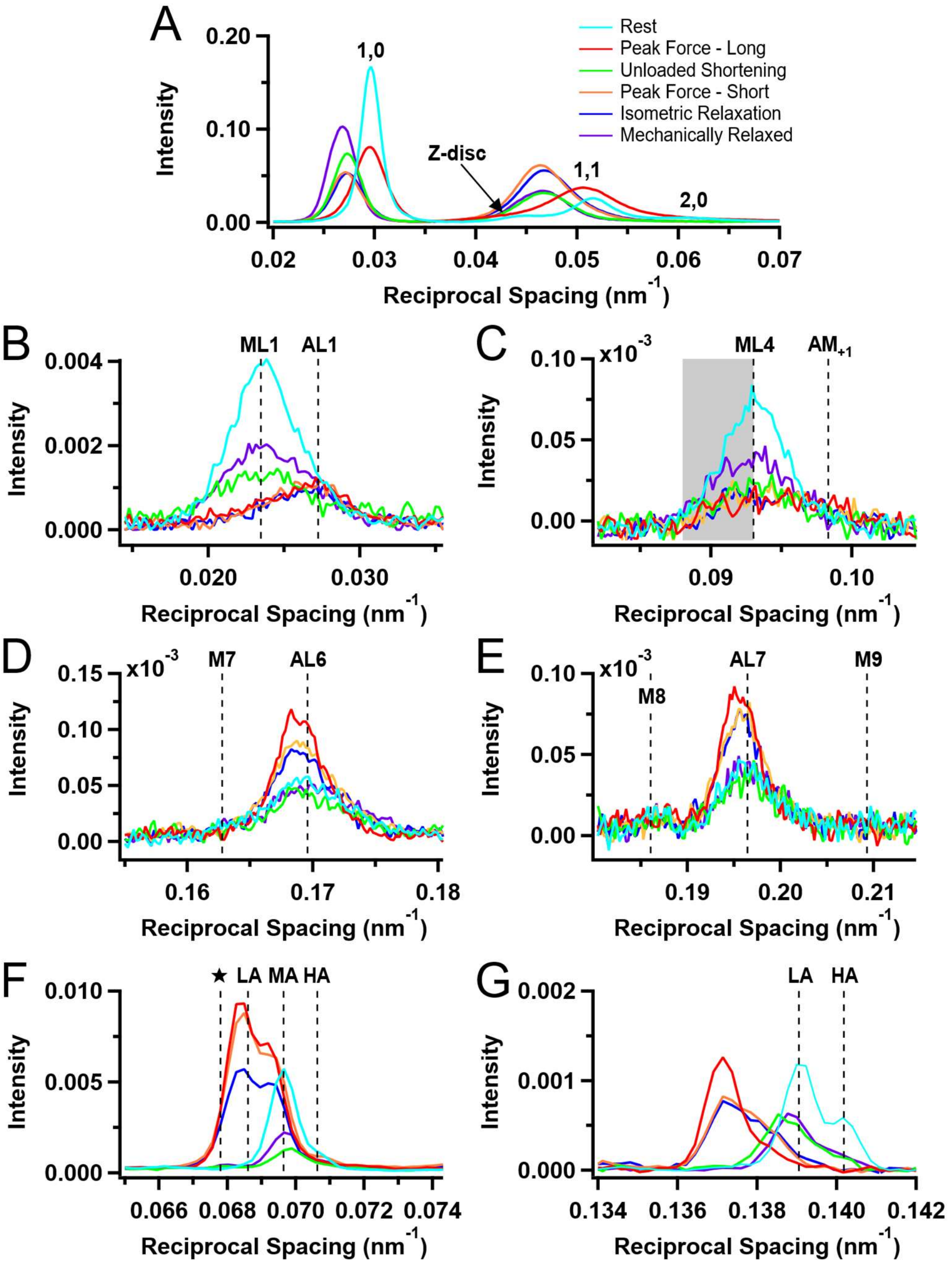

Figure S1 – Intensity distributions of the equatorial, meridional and layer line reflections. (*A*) Equatorial intensity distribution showing the 1,0, 1,1, 2,0 and Z-disc reflections. (*B*) Axial intensity distributions in the region of the ML1 and AL1 layer lines. (*C*) Axial intensity distributions in the region of the ML4 and actomyosin (AM_{+1}) layer lines. I_{ML4} was obtained from the grey-shaded region to avoid any contribution from AM_{+1}. (*D*) and (*E*) Axial intensity distributions in the region of the AL6 and AL7 layer lines, respectively. (*F*) Axial intensity distributions in the region of the third meridional reflection (M3), showing the lower-angle (LA), mid-angle (MA) and higher-angle (HA) sub-peaks and a nearby additional reflection labelled 'star'. (*G*) Axial intensity distributions in the region of the sixth meridional reflection (M6) showing lower-angle (LA) and higher-angle (HA) sub-peaks are labelled. Muscle-to-detector distance, 3.2 m; average of n=4 muscles.

Table S1 - Half-times and rate constants of mechanical and structural signals.

Parameter (N)	Activation $t_{1/2}$ (ms)	Activation P-value vs Force	Unloaded Shortening $t_{1/2}$ (ms)	Unloaded Shortening P-value vs Force	Force Redevelopment $t_{1/2}$ (ms)	Force Redevelopment P-Value vs Force	Relaxation $t_{1/2}$ (ms)	Relaxation P-value vs Force	Slow k_{REL} (s^{-1})	Slow k_{REL} P-value vs Force	Fast k_{REL} (s^{-1})	Fast k_{REL} P-value vs Force
**Force (7)**	16.9 ± 0.4	N/A	3.9 ± 0.2	N/A	9.5 ± 0.3	N/A	28.4 ± 0.9	N/A	5.4 ± 1.0	N/A	104.9 ± 5.5	N/A
**I_{ML1} (7)**	4.3 ± 0.3*	4.49E-07	5.2 ± 0.4†	0.0313	6.0 ± 1.8†	0.1563	39.0 ± 1.7*	0.0002	0.7 ± 1.7	0.0327	22.0 ± 5.2*	8.85E-05
**A_{ML1} (7)**	5.6 ± 0.5*	6.89E-06	4.7 ± 0.4	0.1959	6.7 ± 1.6†	0.1563	34.9 ± 2.6*	0.0125	2.4 ± 3.3	0.3287	33.5 ± 6.6*	8.57E-05
**A_{ML4} (4)**	6.2 ± 0.6*	0.0025	6.5 ± 0.2*	0.0071	6.7 ± 1.6	0.1957	38.5 ± 2.5*	0.0382	0.9 ± 0.1*	0.0280	46.8 ± 11.3*	0.0296
**S_{M6} (4)**	5.2 ± 0.2*	0.0005	4.8 ± 0.2*	0.0368	4.1 ± 0.9*	0.0115	28.6 ± 1.3	0.2586	7.2 ± 3.0	0.8656	91.7 ± 5.1	0.0499
**S_{M11} (3)**	6.3 ± 1.0*	0.0000	4.3 ± 1.1	0.8734	7.2 ± 2.9	0.5878	31.3 ± 4.7	0.7900	5.7 ± 0.9*	0.0396	74.9 ± 13.4	0.1613
**I_{AL2} (3)**	8.8 ± 2.8	0.0000	5.9 ± 1.9	0.4087	7.7 ± 1.2	0.3488	33.7 ± 4.2	0.5051	13.0 ± 6.9	0.3349	103.1 ± 15.3	0.7017
**S_{A13} (4)**	5.5 ± 2.4*	0.0140	3.4 ± 1.1	0.5926	9.6 ± 1.2	0.8579	33.5 ± 5.5	0.2934	35.6 ± 17.4	0.1970	74.0 ± 22.2	0.1008
**I_{M3} (4)**	24.2 ± 0.8*	0.0024	2.1 ± 0.3*	0.0156	11.0 ± 0.3	0.0566	20.0 ± 1.3*	0.0028	38.7 ± 5.7*	0.0081	235.9 ± 8.4*	0.0013
**A_{M3} (4)**	20.8 ± 0.7*	0.0052	2.4 ± 0.3*	0.0258	8.2 ± 0.4*	0.0275	22.3 ± 0.3*	0.0087	35.7 ± 0.9*	0.0125	216.2 ± 6.7*	0.0007
**I_{AL1} (6)**	16.3 ± 2.2	0.745	4.0 ± 0.3	0.9720	8.5 ± 1.6	0.5504	22.3 ± 3.9	0.1030	44.0 ± 16.6	0.0688	N/A	N/A
**I_{AL6} (4)**	11.7 ± 1.5*	0.0695	3.8 ± 0.4	0.9315	9.3 ± 5.1	0.9223	20.6 ± 1.3*	0.0101	46.8 ± 16.9	0.0941	119.4 ± 51.0	0.8944
**I_{AL7} (4)**	11.4 ± 2.1*	0.0388	3.6 ± 0.5	0.6426	15.7 ± 1.9	0.0495	32.4 ± 1.1	0.0681	70.9 ± 44.1	0.2306	84.1 ± 9.1*	0.0037
**$I_{1,1}/I_{1,0}$ (7)**	7.2 ± 0.4*	6.41E-07	N/A	N/A	5.8 ± 0.3*	0.0004	26.9 ± 1.2	0.0662	14.3 ± 1.8*	7.11E-06	72.0 ± 7.4*	5.93E-05

Data were fitted with either an exponential or a sigmoidal curve, and half-time ($t_{1/2}$) was derived from the fits. Measurements of rate constants during isometric relaxation (slow k_{REL}) were calculated as the slope of the linear fit between 120.9 ms and 140.9 ms normalised to the amplitude of a parameter from resting conditions to the first frame after the final electrical stimulation (120.9 ms). Measurements of rate constants during chaotic relaxation (fast k_{REL}) were calculated by exponential fits. Data presented as mean ± S.E.M. N-values provided in brackets beside each parameter. *P<0.05 when comparing a structural parameter against force for half-times and rate constants using Student's paired-samples *t*-test. †P<0.05 when comparing a structural parameter against force for half-times and rate constants using Wilcoxon's signed rank test.

Table S2 – Change in force and structural parameters in key phases of the mechanical protocol.

Parameter (N)	*Key Phase*					
	Rest	Peak Force - Long	Unloaded Shortening	Peak Force - Short	Isometric Relaxation	Mechanically Relaxed
T/T_0 (7)	0.024 ± 0.003	0.94 ± 0.01[a]	0.03 ± 0.01[a,b]	0.91 ± 0.02[a.b,c]	0.96 ± 0.02[a,b,d]	0.003 ± 0.001[a,b,d,e]
I_{ML1} (7)	1	0.15 ± 0.01[a]	0.50 ± 0.03[a,b]	0.14 ± 0.01[a,c]	0.12 ± 0.01[a,c]	0.43 ± 0.01[a,b,d,e]
A_{ML1} (7)	1	0.38 ± 0.01[a]	0.70 ± 0.02[a,b]	0.36 ± 0.01[a,c]	0.34 ± 0.02[a,c]	0.65 ± 0.01[a,b,d,e]
I_{ML4} (7)	1	0.37 ± 0.02[a]	0.44 ± 0.04[a]	0.40 ± 0.03[a]	0.37 ± 0.03[a,c]	0.55 ± 0.01[a,b,d,e]
S_{M6} (4)	7.174 ± 0.0003	7.283 ± 0.001[a]	7.201 ± 0.001[a,b]	7.268 ± 0.001[a,b,c]	7.265 ± ± 0.001[a,b,c]	7.190 ± 0.001[a,b,c,d,e]
S_{M11}[$] (3)	3.916 ± 0.002	3.969 ± 0.001[a]	3.929 ± 0.003[a,b]	3.963 ± 0.001[a,b,c]	3.961 ± 0.001[a,b,c]	3.915 ± 0.002[b,c,d,e]
I_{AL2} (3)	0	1[a]	0.24 ± 0.06[b]	0.77 ± 0.07[a,c]	0.60 ± 0.04[a,b,d]	0.01 ± 0.05[b,d,e]
S_{A13} (4)	2.732 ± 0.001	2.746 ± 0.001[a]	2.731 ± 0.002[a,b]	2.739 ± 0.001[a,c]	2.741 ± 0.001[a,c]	2.724 ± 0.001[a,b,d,e]
I_{M3} (4)	1	2.67 ± 0.05[a]	0.32 ± 0.02[a,b]	2.39 ± 0.06[a,c]	2.15 ± 0.08[a,c]	0.47 ± 0.02[a,b,c,d,e]
I_{AL1} (4)	1	2.18 ± 0.13[a]	0.91 ± 0.08[b]	2.17 ± 0.15[a,c]	1.76 ± 0.12[a,b,c,d]	0.91 ± 0.05[b,d,e]
S_{AL1} (est.) (4)	37.2 ± 0.1	36.8 ± 0.1	36.8 ± 0.1	37.0 ± 0.1	37.2 ± 0.1	37.9 ± 0.1[a,b,c,d,e]
I_{AL6} (4)	1	2.18 ± 0.07[a]	1.04 ± 0.07[b]	1.61 ± 0.08[a,b,c]	1.44 ± 0.06[a,b,c,d]	0.80 ± 0.03[b,c,d,e]
S_{AL6} (4)	5.897 ± 0.003	5.935 ± 0.001[a]	5.901 ± 0.004[b]	5.917 ± 0.002[a,b]	5.920 ± 0.002[a,b]	5.871 ± 0.003[a,b,c,d,e]
I_{AL7} (4)	1	2.21 ± 0.03[a]	1.11 ± 0.06[b]	1.78 ± 0.05[a,c]	1.67 ± 0.06[a,c]	0.88 ± 0.04[a,b,c,d,e]
S_{AL7} (4)	5.090 ± 0.003	5.110 ± 0.001[a]	5.084 ± 0.004[b]	5.101 ± 0.001[a,b,c]	5.106 ± 0.001[c]	5.083 ± 0.002[b,d,e]
$d_{1,0}$ (7)	33.87 ± 0.02	34.04 ± 0.03[a]	36.74 ± 0.16[a,b]	36.91 ± 0.04[a,b]	36.61 ± 0.04[a,b,d]	37.38 ± 0.05[a,b,d,e]
$I_{1,1}/I_{1,0}$ (7)	0.31 ± 0.004	0.83 ± 0.01[a]	0.78 ± 0.03[a]	1.88 ± 0.02[a,b,c]	1.77 ± 0.02[a,b,c,d]	0.61 ± 0.01[a,b,c,d,e]
Volume (nm^3) (4)	3804 ± 6	3567 ± 13[a]	3746 ± 45[b]	3374 ± 13[a,b,c]	3304 ± 10[a,b,c,d]	LA - 3806 ± 25[b,c,d,e]<br>HA – 3744 ± 9[a,b,d,e]
SL (μm)[$] (4)	2.90 ± 0.01	2.69 ± 0.01[a]	2.39 ± 0.03[a,b]	2.13 ± 0.01[a,b,c]	2.13 ± 0.01[a,b,c]	LA – 2.44 ± 0.02<br>HA – 2.32 ± 0.01[a,b,d,e]

Data from the average of 9-10 frames for each key phase (four for unloaded shortening), presented as mean ± S.E.M. N-values are in brackets next to each parameter. Superscripted letters denote significant differences between key phases using a repeated-measures ANOVA with Tukey’s post hoc analyses for parametric data or [$]Friedman’s test with Durbin-Conover pairwise comparisons for non-parametric analyses. [a]P<0.05 when compared to rest. [b]P<0.05 when compared to peak force - long. [c]P<0.05 when compared to unloaded shortening. [d]P<0.05 when compared to peak force - short. [e]P<0.05 when compared to isometric relaxation.

Table S3 – P-values calculated from one-way repeated measures ANOVA's and post-hoc analyses.

Parameter (N)	Main Effect	Rest vs					Peak Force - Long vs				Unloaded Shortening vs			Peak Force - Short vs		Isometric Relaxation vs
		Peak Force - Long	Unloaded Shortening	Peak Force - Short	Isometric Relaxation	Mechanically Relaxed	Unloaded Shortening	Peak Force - Short	Isometric Relaxation	Mechanically Relaxed	Peak Force - Short	Isometric Relaxation	Mechanically Relaxed	Isometric Relaxation	Mechanically Relaxed	Mechanically Relaxed
***T/T_0* (7)**	<1E-16	<1E-16	<1E-16	<1E-16	<1E-16	<1E-16	<1E-16	<1E-16	<1E-16	<1E-16	<1E-16	<1E-16	0.6850	<1E-16	<1E-16	<1E-16
**I_{ML1} (7)**	<1E-16*	4.20E-15	4.60E-15	4.20E-15	4.20E-15	4.20E-15	5.71E-07	0.9988	0.9617	2.64E-07	7.91E-06	2.14E-06	0.4440	0.9996	2.04E-08	2.18E-07
**A_{ML1} (7)**	<1E-16*	1.73E-14	3.57E-12	2.80E-14	1.38E-14	3.52E-13	3.91E-06	0.9841	0.9889	2.96E-05	4.77E-05	1.17E-05	0.9272	0.9998	9.07E-06	1.92E-05
**I_{ML4} (7)**	<1E-16	1.74E-08	4.35E-07	5.59E-10	4.18E-12	7.08E-06	0.4871	0.9999	0.5422	0.0497	0.6408	0.0051	0.6660	0.1634	0.0062	7.92E-05
**S_{M6} (4)**	<1E-16	<1E-16	1.10E-10	<1E-16	<1E-16	1.26E-08	<1E-16	2.74E-05	1.20E-05	<1E-16	<1E-16	<1E-16	0.0006	1	<1E-16	<1E-16
**$S_{M11}$$ (3)**	1.28E-10	<1E-16	1.20E-06	<1E-16	<1E-16	0.2801	<1E-16	8.18E-08	3.16E-07	<1E-16	5.33E-09	1.35E-09	2.09E-08	0.7175	<1E-16	<1E-16
**I_{AL2} (3)**	<1E-16	3.30E-05	0.0662	3.22E-06	0.0011	0.9307	1.23E-05	0.1380	0.0034	4.69E-05	6.60E-07	0.2619	0.2006	0.0015	1.08E-05	0.0039
**S_{A13} (4)**	1.16E-09*	1.98E-05	0.0162	1.36E-06	0.0019	8.99E-05	0.0001	0.2580	0.5137	3.14E-08	0.0004	0.0019	0.8188	1.0000	8.41E-07	2.35E-07
**I_{M3} (4)**	<1E-16	<1E-16	<1E-16	6.13E-12	1.01E-09	1.00E-12	<1E-16	0.4811	0.3514	<1E-16	<1E-16	4.44E-12	2.247E-05	0.9785	<1E-16	4.03E-12
**I_{AL1} (4)**	5.45E-10*	2.67E-05	0.0505	1.91E-05	0.0004	0.6663	5.76E-07	1.0000	0.0030	1.70E-06	3.50E-07	2.03E-06	0.3779	0.0017	5.79E-07	1.54E-05
**S_{AL1} (est.) (4)**	3.76E-08	0.7949	0.4347	0.9999	0.9964	0.0455	0.8594	0.5339	0.8774	3.05E-05	0.3627	0.5993	0.0002	0.9996	0.0041	0.0016
**I_{AL6} (4)**	<1E-16	2.54E-08	0.9270	1.16E-05	2.72E-05	0.0563	1.62E-08	4.57E-07	2.15E-07	6.05E-11	1.41E-06	1.62E-06	0.0091	0.0012	1.59E-08	2.66E-09
**S_{AL6} (4)**	<1E-16	0.0004	0.7127	0.0476	0.0103	0.0202	9.68E-06	0.0004	0.0237	2.38E-10	0.1675	0.0994	9.63E-08	0.7904	1.63E-07	2.64E-07
**I_{AL7} (4)**	<1E-16	9.33E-11	0.7354	8.48E-08	2.63E-05	0.0017	2.89E-08	0.1363	0.0695	<1E-16	1.12E-05	4.96E-06	0.0039	0.8074	5.63E-10	5.40E-08
**S_{AL7} (4)**	4.52E-07*	0.0008	1.0000	0.0380	0.0968	0.9001	4.32E-06	9.39E-07	0.1887	6.18E-06	0.0024	0.0072	0.8159	0.9516	0.0004	0.0008
**$d_{1,0}$ (7)**	<1E-16*	0.0012	4.80E-15	4.20E-15	4.20E-15	4.20E-15	5.60E-15	4.20E-15	4.20E-15	4.2E-15	0.9803	0.8592	0.0966	4.20E-15	0.0011	8.02E-07
**$I_{1,1}/I_{1,0}$ (7)**	<1E-16*	<1E-16	1.34E-07	<1E-16	<1E-16	<1E-16	0.9956	<1E-16	<1E-16	3.17E-11	<1E-16	<1E-16	0.0027	0.2930	<1E-16	8.02E-07
**Volume (nm^3) - HA (4)**	<1E-16*	<1E-16	0.1329	<1E-16	<1E-16	0.0016	4.01E-11	<1E-16	<1E-16	3.63E-08	<1E-16	<1E-16	0.9129	<1E-16	<1E-16	<1E-16
**Volume (nm^3) - LA (4)**	<1E-16*					0.8149				8.94E-11			0.0143		1.79E-13	3.36E-14
**SL (μm)$ (4)**	<1E-16	<1E-16	<1E-16	<1E-16	<1E-16	<1E-16	8.46E-11	<1E-16	<1E-16	<1E-16	1.53E-09	1.15E-09	0.6040	0.0914	1.42E-11	3.65E-12

P-values correspond to significant differences derived from post-hoc analyses in Table S3. $Friedman's test with Durbin-Conover pairwise comparisons for non-parametric analyses. *Greenhouse-Geisser sphericity correction for main effects.

Dataset S1 (separate file). Data associated with figures 1-6 of the main article file, and Table S1 of the supplementary file.

SI References

1. T. Narayanan, *et al.*, Performance of the time-resolved ultra-small-angle X-ray scattering beamline with the Extremely Brilliant Source. *J. Appl. Crystallogr.* **55**, 98–111 (2022).

2. T. Narayanan, W. Chèvremont, T. Zinn, Small-angle X-ray scattering in the era of fourth-generation light sources. *J. Appl. Crystallogr.* **56**, 939–946 (2023).

3. M. Sztucki, SAXSutilities2: a graphical user interface for processing and analysis of Small-Angle X-ray Scattering data. (2021). https://doi.org/10.5281/zenodo.5825707. Deposited 21 September 2021.

4. C. A. Schneider, W. S. Rasband, K. W. Eliceiri, NIH Image to ImageJ: 25 years of image analysis. *Nat. Methods* **9**, 671–675 (2012).

5. H. E. Huxley, A. R. Faruqi, M. Kress, J. Bordas, M. H. J. Koch, Time-resolved X-ray diffraction studies of the myosin layer-line reflections during muscle contraction. *J. Mol. Biol.* **158**, 637–684 (1982).

6. M. Caremani, *et al.*, Dependence of thick filament structure in relaxed mammalian skeletal muscle on temperature and interfilament spacing. *J. Gen. Physiol.* **153**, e202012713 (2021).

7. C. Hill, E. Brunello, L. Fusi, J. G. Ovejero, M. Irving, Myosin-based regulation of twitch and tetanic contractions in mammalian skeletal muscle. *eLife* **10**, e68211 (2021).

8. C. Hill, E. Brunello, L. Fusi, J. G. Ovejero, M. Irving, Activation of the myosin motors in fast-twitch muscle of the mouse is controlled by mechano-sensing in the myosin filaments. *J. Physiol.* **600**, 3983–4000 (2022).

9. J. Bordas, *et al.*, Extensibility and symmetry of actin filaments in contracting muscles. *Biophys. J.* **77**, 3197–3207 (1999).

10. W. Ma, H. Gong, T. Irving, Myosin Head Configurations in Resting and Contracting Murine Skeletal Muscle. *Int. J. Mol. Sci.* **19**, 2643 (2018).

11. H. Iwamoto, T. Suzuki, T. Fujisawa, Time-resolved two-dimensional X-ray diffraction study of the effect of shortening on activation of contracting skeletal muscle. *Pflüg. Arch.* **439**, 646–649 (2000).

12. T. Tamura, J. Wakayama, K. Inoue, N. Yagi, H. Iwamoto, Dynamics of Thin-Filament Activation in Rabbit Skeletal Muscle Fibers Examined by Time-Resolved X-Ray Diffraction. *Biophys. J.* **96**, 1045–1055 (2009).

13. M. Kress, H. E. Huxley, A. R. Faruqi, J. Hendrix, Structural changes during activation of frog muscle studied by time-resolved X-ray diffraction. *J. Mol. Biol.* **188**, 325–342 (1986).

14. B. Kiss, *et al.*, Nebulin stiffens the thin filament and augments cross-bridge interaction in skeletal muscle. *Proc. Natl. Acad. Sci. U. S. A.* **115**, 10369–10374 (2018).

15. The Jamovi Project, (2024). Deposited 2024.
